# Triggering cell death in cancers using self-illuminating nanocomposites

**DOI:** 10.3389/fchem.2022.962161

**Published:** 2022-09-15

**Authors:** Tijana Rajh, Tamara Koritarov, Ben Blaiszik, Syeda Fatima Z. Rizvi, Vani Konda, Marc Bissonnette

**Affiliations:** ^1^ Center for Nanoscale Materials, Argonne National Laboratory, Argonne, IL, United States; ^2^ School of Molecular Sciences, Arizona State University, Tempe, AZ, United States; ^3^ Department of Medicine, The University of Chicago Medicine, Chicago, IL, United States

**Keywords:** TiO_2_, nanocomposites, luciferase, bioluminescence, extracellular ATP, cancer, apoptosis, real-time confocal microscopy

## Abstract

Bioinspired photocatalysis has resulted in efficient solutions for many areas of science and technology spanning from solar cells to medicine. Here we show a new bioinspired semiconductor nanocomposite (nanoTiO_2_-DOPA-luciferase, TiDoL) capable of converting light energy within cancerous tissues into chemical species that are highly disruptive to cell metabolism and lead to cell death. This localized activity of semiconductor nanocomposites is triggered by cancer-generated activators. Adenosine triphosphate (ATP) is produced in excess in cancer tissues only and activates nearby immobilized TiDoL composites, thereby eliminating its off-target toxicity. The interaction of TiDoL with cancerous cells was probed *in situ* and in real-time to establish a detailed mechanism of nanoparticle activation, triggering of the apoptotic signaling cascade, and finally, cancer cell death. Activation of TiDoL with non-cancerous cells did not result in cell toxicity. Exploring the activation of antibody-targeted semiconductor conjugates using ATP is a step toward a universal approach to single-cell-targeted medical therapies with more precision, efficacy, and potentially fewer side effects.

## 1 Introduction

Recent progress in advanced medical therapies is focused on targeted therapies with a single-cell level resolution. They hold the promise of reducing off-target drug effects by directing therapeutic agents exclusively to localized diseased tissue (O’Brien et al., 2007; Li, 2014; Petros and DeSimone, 2010; Yang et al., 2018; Schmidt et al., 2020; Jan et al., 2021; Briolay et al., 2021). Several factors are important for cell-targeted therapies among which prevention of the premature release of toxic remedies is one of the most important ones. Several drug delivery systems activated by heat, magnetic fields, ultrasound, or light are being explored (Mura et al., 2013; Briolay et al., 2021; Han and Choi, 2021). Another attractive approach to localized therapies is the use of molecules that are produced by the disease itself as triggers that activate the therapy (LaVan et al., 2003). So far, this approach has been used indirectly for carriers that rely on pH change across the liposome bilayer (Maurer-Spurej et al., 1999; Lei et al., 2017) or pathophysiological abnormalities of the vascular system in tumors (EPR effect) (Allen and Cullis, 2004; Lei et al., 2017) that passively accumulate and retain the drug at diseased targets. We take a new approach that takes advantage of the active release of ATP in the extracellular environment of tumors (Stagg and Smyth, 2010; Vultaggio-Poma et al., 2020). One of the most prominent metabolic alterations in cancer cells is the increase in aerobic glycolysis and the dependency on the glycolytic pathway for ATP generation, known as the Warburg effect (Pelicano et al., 2006). Many cancers produce a significant amount of ATP through a glycolic Warburg pathway and maintain high levels of ATP through avid consumption of glucose even during the execution of apoptosis (Oberdanner et al., 2002; Kim and Dang, 2006; Vultaggio-Poma et al., 2020). This highly energetic molecule is accumulated in the tumor interstitium, while it is basically undetectable in healthy tissues (Pellegatti et al., 2008). The excess ATP was previously used for imaging cancers deep in the tissues or to induce luminescence of quantum dots (Kim et al., 2015).

TiO_2_ nanoparticles exhibit exceptional photoreactivity (Rajh et al., 2014) and can be functionalized to bind multiple biomolecules to an individual particle (Endres et al., 2007) and initiate photochemical reactions within the cell environment (Paunesku et al., 2003). Their surface modification with enediol molecules extends their light absorption to the visible and near-infrared region of the light spectrum, overcoming the biggest obstacle to the use of TiO_2_ nanoparticles for applications in phototherapy (Supplementary Figure S1) (Rajh et al., 2002). It was shown that enediol-modified TiO_2_ nanoparticles are highly active under visible light (Liu et al., 2006; Tachan et al., 2014) and have the ability to create reactive radical species upon illumination with visible light (Dimitrijevic et al., 2009). However, the small penetration depth of light in the body still limits the use of semiconductors in photodynamic therapy, restricting the targets to the skin and the lining of a lumen.

**SCHEME 1:**
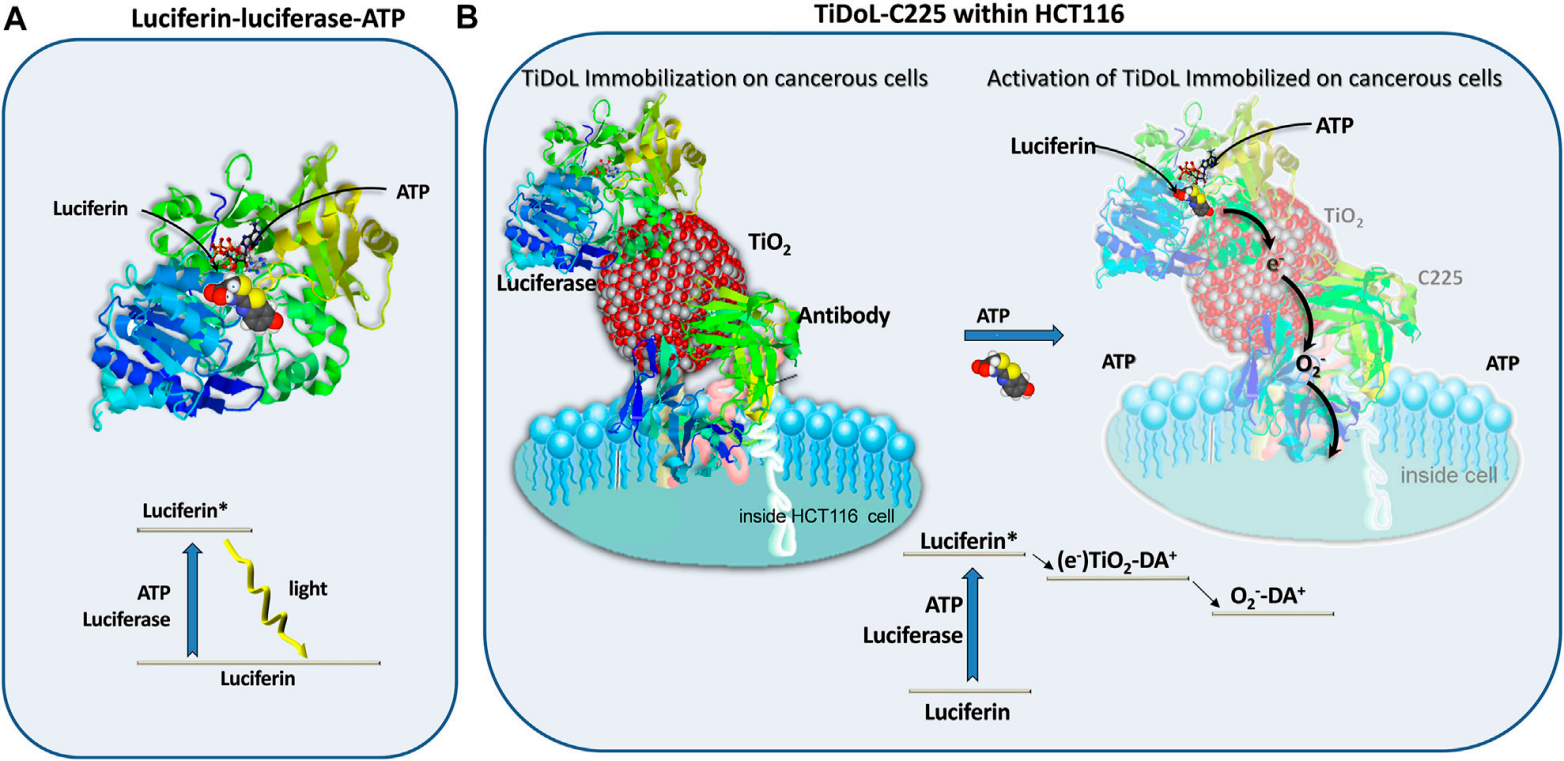
Silent features of firefly luciferase catalyzed excitation and luminescence of luciferin in the presence of ATP **(A)** and modification of the excitation mechanism to power membrane-immobilized semiconductor particles in tumors *in situ* resulting in radical species such as superoxide radicals (O_2_^−^·) capable of initiating the apoptotic signaling cascade within a neighboring cancerous cell **(B)**.

In this study, we describe a different approach; instead of delivering the light to the tumor, we take advantage of the presence of the highly energetic molecular source ATP in the extracellular space of the cancerous tissue to create superoxide radicals (O_2_^−^·) capable of initiating the apoptotic signaling cascade (Chauhan et al., 2003; Dimitrijevic et al., 2009). Just like ATP activates luciferase-mediated luciferin emission of light (Scheme 1A), a mechanism used by fireflies to create their own light, ATP can power TiO_2_ nanocomposites and directly generate electrons capable of a further generation of superoxide radicals O_2_^−^· (Scheme 1B). Superoxide is rather unreactive and its diffusion distance ranges from 0.5 to a few μm, largely controlled by the concentration of superoxide dismutase and nitric oxide radicals (compared to <2 nm for OH⋅ radicals and <250 nm of singlet oxygen) (Panus et al., 1993; Mikkelsen and Wardman, 2003). As excess ATP is local to tumor regions, photocatalytically produced superoxide radicals are formed within the tumor microenvironment and can diffuse to adjacent cell mitochondria and induce an apoptotic cell-signaling cascade (Chauhan et al., 2003).

In this work, we show that the sensitization of TiO_2_ with luciferase enables ATP activation of TiO_2_ nanoparticles and mediates an apoptotic cascade within HCT 116 colon cancerous cells. The elevated level of extracellular ATP within the tumor microenvironment is responsible for the localization of composite activity and formation of superoxide radicals within the tumor regions. We demonstrate that nanocomposites do not show any activity within noncancerous immortalized cells YAMC (young adult mouse colonocytes). We explore the strategy for enhancing nanocomposites’ retention within the extracellular space, by coupling luciferase-TiO_2_ nanocomposites (TiDoL) to the C225 antibody capable of recognizing and binding to receptors uniquely expressed on the HCT 116 surface. Antibody-immobilized nanocomposites confine the formation of radical species to the surface of HCT 116 cells and enhance the efficiency of interaction between superoxide radicals O_2_^−^· and nearby cancerous cells. In this way, the release of superoxide radicals becomes doubly regulated by 1) the level of ATP production in cancerous cells, and 2) the positioning of TiO_2_ nanoparticles near cancerous cells using cell-specific antibodies, limiting their activity exclusively to cancerous tissues and therefore mitigating non-systemic toxicity.

## 2 Materials and methods

### 2.1 TiO_2_ sample preparation

Five nm TiO_2_ nanoparticles in water were prepared by the drop-wise addition of TiCl_4_ to cooled water under vigorous steering as described previously (Rajh et al., 1996). Nanoparticles were deposited on ITO-coated glass slides *via* electrophoretic deposition (EPD) in aqueous solution at a voltage of 20 V and ca. 5 µA current. After deposition, the TiO_2_-coated electrodes were sintered at 400°C for 1 h. Following the sintering process, a lead wire was attached to the sample and conduction between the wire and ITO was achieved by the addition of conductive silver paint. The surface of the TiO_2_ nanoparticle films was modified by ∼12 h of immersion in 10 mg/ml DOPAC solution in 10 mM MES buffer solution at pH 6.1 in the dark. Following functionalization, the carboxyl group of the surface-tethered Dopac was activated by reaction with 50 mM 1-Ethyl-3-[3-dimethylaminopropyl]carbodiimide (EDC) and 44 mM N-hydroxysulfosuccinimide (sulfo-NHS) in MES buffer at pH 6.1 for 2 h. Any remnant EDC-sulfo-NHS was quenched by reaction with excess mercaptoethanol and washed further with MES buffer. After washing, varying amounts of a luciferase enzyme were added to a vial containing the electrodes and MES buffer and allowed to react overnight in a dark refrigerator at 4°C. TiO_2_ nanoparticles in phosphate buffer (10 mM PBS, pH 7) were prepared by rapid transition of positive particle charge at pH 3 to negative particle charge at pH 9 by injection of 1M LiOH to TiO_2_ solution and subsequent dialysis against 10 mM PBS.

### 2.2 Chemically induced electrochemical testing

Current measurements were performed on the electrodes in a solution of 10 mM MES buffer (pH 6.1) and 5 mM hydroquinone purged with Ar. No bias voltage was applied, and the reference electrode was Ag/AgCl. The current was allowed to stabilize for 30 s before the luciferin and ATP substrate were injected. For a control film of TiO_2_ modified with Dopac, only a nominal change in current was detected when the luciferin-ATP substrate was injected. Experiments were replicated two times.

### 2.3 Monitoring light emission from functionalized TiO_2_ nanoparticles

Light emission from the functionalized TiO_2_ nanoparticles in the solution and nanoparticle films was monitored using a Horiba Nanolog Spectrofluorometer. No light emission was observed for any control sample (TiO_2_ and TiO_2_-Dopac nanoparticles) in the spectrofluorometer after treatment with 100 µl of luciferin/ATP substrate. However, when functionalized TiO_2_-DOPAC-luciferase films were treated with 100 µl of luciferin/ATP, an emitted light was observed. The decay kinetics and the emission spectrum were both monitored. Experiments were replicated two times.

### 2.4 Synthesis of self-illuminating nanoconjugates

We synthesized self-illuminating semiconductor nanoconjugates of titanium dioxide—Dopac—luciferase (TiDoL) by covalent linking of luciferase (*Photinus pyralis* (*firefly*)) with an equimolar particle concentration of 5 nm-TiO_2_ nanoparticles through a conductive 3,4-Dihydroxyphenylacetic acid (Dopac) linker. To link negatively charged TiO_2_ nanoparticles (in 10 mM phosphate buffered saline, PBS, pH 7) to luciferin that also has an overall negative charge (formal negative charge of −7.0), a small negatively charged linker Dopac (20 fold excess) was first incubated with luciferin to position negatively the charged carboxyl group in the positive pockets of luciferin (Supplementary Figure S2, five large positive pockets, mainly composed of amino group terminated arginine and lysine) followed by EDS/sulfo-NHS conjugation of Dopac to amino groups of luciferase. TiO_2_ nanoparticles were then incubated with (comprehensively washed) Dopac-modified luciferase. Due to their high degree of surface curvature, bare TiO_2_ nanoparticles have surface atoms in a distorted crystalline environment and under-coordinated geometry (Rajh et al., 1999). These under-coordinated sites at the surface of small TiO_2_ nanoparticles exhibit high reactivity toward bidentate coordination with oxygen-containing ligands enabling their seamless coupling to small molecules such as Dopac. Bidentate binding of Dopac to under-coordinated sites results in reconstruction of the surface atoms of TiO_2_ nanoparticles to thermodynamically stable octahedral geometry. This, in turn, introduces new electronic states in the mid-gap region of TiO_2_ nanoparticles that originate from Dopac HOMO and lead to enhanced optical properties of the nanoconjugate in the visible region of the spectrum (Rajh et al., 2002; Rego and Batista, 2003). As a result, only successful coupling of bare TiO_2_ nanoparticles to DOPAC-modified luciferase will result in the electronic interaction manifested by the appearance of the visible absorption band (Paunesku et al., 2003; Rajh et al., 2004) of the nanoconjugate (TiO_2_–Dopac–Luciferase, TiDoL). The appearance of visible absorption and the coupling were monitored by UV absorption spectroscopy and the measurements of zeta potential and electrophoretic mobility using laser Doppler micro-electrophoresis (Supplementary Figure S1; Supplementary Table S1). Detailed linking procedures are as follows:

#### 2.4.1 TiO_2_ functionalization/TiDoL

A total of 10 μl of 10 mg/ml Dopac (Sigma-Aldrich, St. Louis, MO, United States) was added to the 250 µl of stock 8.33 µM luciferase (Sigma-Aldrich, St. Louis, MO, United States) under nitrogen and incubated for 30 min. Then 20 µl of already mixed 0.5 ml of 10 mg/ml 1-Ethyl-3-[3-dimethylaminopropyl] carbodiimide hydrochloride (EDC) (Sigma-Aldrich, St. Louis, MO, United States), and 0.5 ml of 10 mg/ml N-Hydroxysuccinimide (NHS) (Sigma-Aldrich, St. Louis, MO, United States) solution was added to the luciferase solution under nitrogen and incubated for 2 h at room temperature under nitrogen. Then, the solution was washed four times with phosphate-buffered saline (PBS) (Sigma-Aldrich, St. Louis, MO, United States) using a centrifuge purification system. After the last wash, the solution was concentrated at 100 µL, and 200 µl of 10 µ of 5 nm TiO_2_ was added. A faint yellow color appeared upon the addition of TiO_2_ as a result of the formation of a charge transfer complex between TiO_2_ and Dopac linked to luciferase confirming a successful coupling reaction. When the equimolar concentration of Dopac was used for modification of luciferase, only partially conjugated TiDoL was obtained as observed by UV/vis absorption spectroscopy. This preparation leads to a final concentration of 6.2 µM TiDoL with an excess of 3.3 μM luciferase. This TiDoL solution was used both as synthesized and with the addition of 25 µl of Dopac to enhance the absorption of luciferase-mediated luciferin emission.

#### 2.4.2 Functionalization of TiDoL with C225 antibody/TiDoL-C225

TiDoL was further modified with an antibody such as C225. For that purpose, 10 μl of 10 mg/ml Dopac (Sigma-Aldrich, St. Louis, MO, United States) was added to 125 µl of 13.3 µM of an anti-EGFR monoclonal antibody, C225 (2 mg/ml Cetuximab/Erbitux, Eli Lilly, New York, NY, United States) under nitrogen. Then 20 µl of already mixed 0.5 ml of 10 mg/ml EDC and 0.5 ml of 10 mg/ml NHS solution was added under a nitrogen atmosphere. The solution was incubated for 2 h at room temperature under nitrogen. Then, the solution was washed four times with PBS using centrifuge purification. After the final wash, the final volume was adjusted to 125 and 300 µl of 6.2 µM TiDoL was added (or TiO_2_ for preparation of TiO_2_-C225). This preparation makes a final TiDoL-C225 solution with a concentration of 4.15 µM with a 0.28 µM concentration of free C225, which is approximately 5% excess.

#### 2.4.3 Functionalization of TiDoL and TiDoLC225 with alizarin

TiDoL and TiDoL-C225 were modified with alizarine for monitoring nanocomposite fluorescence. A total of 25 µl of TiDoL (TiDoL-C225) was incubated with 372.5 µl of PBS and 2.5 µl of 6 mM alizarine. Pale pink color of the solution was developed after sonication for 5 min.

### 2.5 Particle characterization

The *Zetasizer* (Malvern Zetasizer Nano-ZS, Malvern, United Kingdom) was used to measure the size, zeta potential (surface charge), and mobility of TiO_2_ and TiDoL samples (Supplementary Table S1). Samples were placed in an optical cell equipped with gold electrodes. Dynamic light scattering was measured in the cell using a 633 nm light laser cell with an applied potential and zeta potential, mobility, conductivity, and the size of nanoconjugates were obtained.

### 2.6 Cell culture

The HCT116 human colon cancer cell line with an overexpression of epidermal growth factor (EGF) receptors (ATCC, 2013) was grown in McCoy’s 5a Medium Modified (Gibco, Grand Island, NY, United States), with fetal bovine serum (Gibco, Grand Island, NY, United States) added to a final concentration of 10%, and penicillin–streptomycin (Sigma-Aldrich, St. Louis, MO, United States) added to a final concentration of 5% ay 37°C. The young adult mouse colonocyte (YAMC) cell line (provided by the University of Chicago Medical Center, Chicago IL, United States, 2015) was derived from the *Immortomouse*, a transgenic animal containing a temperature-sensitive T Ag under the control of an IFN-γ-dependent promoter. YAMC cells proliferate under permissive conditions of 33°C in the presence of 5 U/ml IFN-γ (PeproTech). YAMC cells were cultured in RPMI 1640 containing 5% FBS, 2 mM l-glutamine, penicillin/streptomycin, 5 U/ml IFN-γ, and N-2 supplement (Invitrogen Life Technologies). CT 26 fibroblast cells (ATCC, 2014) were grown in the same media with 10% FBS at 37°C. Cells were cultured under nonpermissive conditions for at least 24 h before experiments, and for the duration of experimentation. The cells were subcultured when they are grown at 70–90% confluence. An optical upright microscope (VWR VistaVision, Radnor, PA, United States) equipped with a camera (Carl Zeiss AxioCam CM1, Oberkochen, Germany) was used to examine the cell cultures and monitor the progress daily. A randomized block design is used for *in vitro* and *in situ* experiments. The cells were divided into eight or four identical blocks, and experimental procedures on each block were assigned randomly.

#### 2.6.1 Cell staining

Every cellular dye was applied with a specific staining protocol described by the manufacturer. The cellular dyes were added to the growth medium incubated and washed with a growth medium and HBSS. Cell membranes of human colorectal cancer cells, including the HCT116 cell line, express multiple glycoprotein ligands (Tomlinson et al., 2000; Nagano et al., 2011) allowing the use of WGA for sensitive imaging of cytoplasmic membranes in HCT116 cell line (Chazotte, 2011; Wang et al., 2012). In addition, fluorescent *WGA* was also used to *stain* the Golgi apparatus (Allen et al., 1989; Chazotte, 2011) as cisternae at the trans face of Golgi membrane stacks participate in glycosylation of proteins that also bind WGA (Elmouelhi et al., 2008). Therefore, WGA (Invitrogen, Grand Island, NY, United States) was used for staining the cell membrane and Golgi apparatus in a fluorescent green color (Alexa 488). Mitotracker red (Invitrogen, Grand Island, NY, United States), a cationic red-fluorescent dye, binds negatively charged mitochondria in live cells and was used for labeling mitochondria with red fluorescence. It possesses a reactive chloromethyl group that forms a covalent bond with free thiols in proteins and peptides, retaining labeling thiol-containing proteins even after mitochondrial depolarization. Mitosox Red (Invitrogen, Grand Island, NY, United States) was a cellular stain that was used to image the superoxide radicals within the cell environment. The nucleus of the cellular cultures was stained with Hoechst (Invitrogen, Grand Island, NY, United States). Hoechst fluorescence is significantly enhanced in condensed chromatin and is used for motoring pyknotic nuclei. Alizarin was used to monitor TiO_2_ nanoparticles within the cell environment. The Zeiss LSM 510 Meta confocal microscope (Carl Zeiss, Oberkochen, Germany) was used to image colocalization in stained cell cultures. All experiments were replicated three times and 200 cells were examined for statistics.

### 2.7 Real-time monitoring of the activity of self-illuminating TiDoL nanoparticles within the cell environment

HCT116 colon cancer cells were grown in 4-well or 8-well plates. For 8-well plates, the final volume of the solution containing all reactants and cell culture media, Hanks Balanced Salts Solution (HBSS), or PBS were 300 µl, and for 4-well plates, the amounts were doubled to a final volume of 600 µl. TiDoL nanoconjugates and controls (luciferase, TiO_2_—Dopac, and unconjugated TiO_2_—Dopac mixed with luciferase, all in the same 600 nM concentration) were incubated with cells in the presence of ATP (20 μM). The Zeiss LSM 510 Meta confocal microscope (Carl Zeiss, Oberkochen, Germany) was used to visualize cell culture changes in real-time. After 20 min of incubation, an aliquot of luciferin (15 μM final concentration) was injected into the well and the response of the cells to the addition of luciferin was monitored using the transmission mode of a confocal microscope with minimal light exposure that does not perturb the cells in the absence of luciferin injection (Figure 2A, time 0 min). The movement of the cells was recorded for 5 min before injection and 1 h after injection. The injections were repeated until budding of the cells was observed. The movies were made by taking time series photographs with the confocal microscope and enabled continuous monitoring of the effects of luciferin therapy on the cellular level. For investigating the effects of the presence of the antibody, TiDoL-C225 nanoconjugates and controls (luciferase, C225-Dopac and C225-TiO2-Dopac, all in the same 400 nM concentration) were incubated with cells in the presence of ATP (20 μM). Experiments were replicated three times and 200 cells were examined for statistics.

### 2.8 *In vivo* study of the TiDoL/TiDoL-C225 xenograft model in nude mice

An *in vivo* study with nude mice was carried out in the animal facility at the University of Chicago. All the studies were conducted in compliance with the Institutional Animal Care and Use Committee’s requirements for the care and use of laboratory animals in research. A total of 16 nu/nu mice (6 weeks old, male) were implanted with HCT116 cells [3 × 10 (Jan et al., 2021) cells per flank] to induce xenograft tumors. TiDoL, TiDoL-C225, or C225 alone were injected into the tumors at 4 weeks (1.75 μg/g TiDoL, 50 μl injection and 1.25 μg/g TiDoL-C225, 50 μl injection). Half the mice in each group (allocation of animals to the group was random) received IV luciferin × 2 (intraperitoneally, 15 mg/ml in PBS, 10 μl/g of body weight) and the remainder received vehicle. The tumors were measured every 2–3 days for 4 weeks. The tumor volume was calculated using expression volume = (length × width) (Li, 2014)/2 (mm^3^). All animals were included in the analysis. Tumor volumes were plotted and fitted to an exponential function of growth using OriginLab. The average values of tumor volumes (Mean) and standard deviations (SD) were obtained using OriginLab and computed by the following expressions:

$$\begin{array}{ccc} \text{Mean}=\sum_{i=0}^{n}x_{i} & \text{SD}=\sqrt{\sum_{i=1}^{n}\frac{{\left(x_{i-}x¯\right)}^{2}}{d}} & \text{where d}=n-1 \end{array}.$$

In the graphs presented in Figure 5, the values were presented as mean (average) values and error bars as standard deviations. Standard deviation values were similar for each group. Histology was performed on tumors surgically removed from sacrificed mice 26 days after treatment, fixed in 3.7% formaldehyde, and embedded in paraffin blocks. Slices were stained with H&E under standard conditions and evaluated for pathological markers by transmission optical and laser confocal microscopy using white light and two color laser excitation (560 and 633 nm light), respectively (Zeiss LSM 510 Meta confocal microscope).

### 2.9 Statistical analysis

*In vivo* experiments’ data were presented as the average of six tumor measurements and each experiment was carried out at least by duplicates. *In vivo* data were expressed as means ± S.D. (standard deviation). Statistical analysis was performed by using Student’s *t*-test employing six independent data points. The criterion for statistical significance was *p* < 0.05. We used Excel (T.TEST function) for the determination of the *p*-value.

## 3 Results and discussion

### 3.1 Preparation of adenosine triphosphate-powered nanocomposites

**FIGURE 1:**
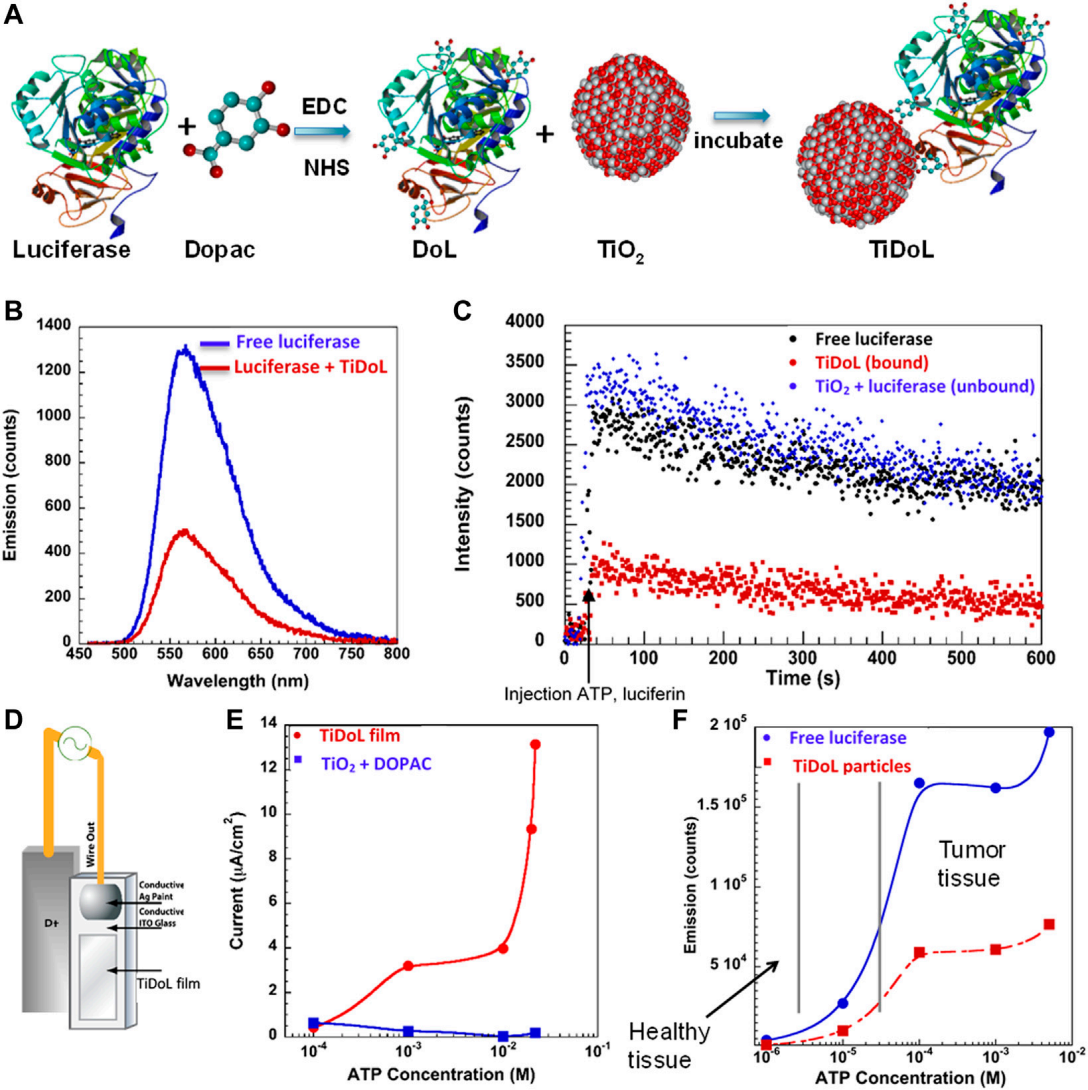
**(A)** Synthesis of self-powering TiDoL nanoparticles. In the first step, 3,4 dihydroxyphenylacetic acid (Dopac) was incubated with luciferase and subsequently reacted using EDC/NHS chemistry. Dopac-labeled luciferase (DoL) was then incubated with 5 nm TiO_2_ nanoparticles to result in luciferase covalently linked to TiO_2_ nanoparticles *via* a conductive linker (TiDoL). **(B)** Emission of free luciferase activated by luciferin in solution (blue) is quenched by TiDoL nanoparticles (red). **(C)** Kinetics of light emission of free luciferase (10 nM) and luciferin (40 μM) in solution (black); free luciferase and luciferin in the presence of, but not conjugated to, an equimolar concentration of TiO_2_-Dopac (10 nM particles) (blue); and TiDoL nanoparticles (10 nM) and luciferin (40 μM) in solution (red). **(D)** Schematics of experimental luciferin induced current measurement setup in conjunction with **(E)** current generated by the luciferase-mediated conversion of luciferin in TiDoL films as a function of ATP concentration. Current measurements were performed on the electrodes in a solution of 10 mM MES buffer (pH 6.1) and 5 mM hydroquinone purged with Ar. **(F)** Bioluminescence emitted by the luciferase-mediated catalysis of luciferin at various concentrations of ATP. Minimal bioluminescence is emitted at ATP concentrations found in healthy tissue (low micromolar levels) for both unconjugated luciferase (blue) and luciferase that has been conjugated to TiO_2_-Dopac nanoparticles (red). Significant bioluminescence and bioluminescence quenching by TiDoL nanoparticles are detected at ATP concentrations present in tumor tissue (low millimolar levels).

We synthesized self-illuminated semiconductor nanoconjugates (TiDoL) by covalent linking of *Photinus pyralis* (firefly) luciferase with 5 nm TiO_2_ nanoparticles through a conductive 3,4-Dihydroxyphenylacetic acid (Dopac) linker (Figure 1A). To link negatively charged TiO_2_ nanoparticles (formal negative charge of −7.0 mV, Supplementary Table S1) to luciferase that has an overall negative charge (−5.82 mV), small molecule linker Dopac was first incubated with luciferase to position the negatively charged carboxyl group within the positive pockets of the protein (Supplementary Figure S2, five large positive pockets, mainly composed of amino group-terminated arginine and lysine). Following incubation, EDS/sulfo-NHS conjugation was used to couple the Dopac carboxyl group to an amino group of luciferase. TiO_2_ nanoparticles were then added and incubated with Dopac-modified luciferase. Due to their high degree of surface curvature, bare 5 nm TiO_2_ nanoparticles have surface atoms in under-coordinated geometry (Rajh et al., 1999). These under-coordinated sites at the surface of small TiO_2_ nanoparticles exhibit high reactivity toward bidentate coordination with oxygen-containing ligands enabling their coupling to small molecules such as Dopac. Bidentate binding of Dopac to under-coordinated sites results in the reconstruction of the surface atoms of TiO_2_ nanoparticles to thermodynamically stable octahedral geometry (Rajh et al., 2002; Rajh et al., 2014). This, in turn, introduces new electronic states in the mid-gap region of TiO_2_ nanoparticles, creating a hybrid nanoparticle with Dopac HOMO and TiO_2_ LUMO, leading to enhanced optical properties of the nanoconjugate in the visible region of the spectrum (Rajh et al., 2002; Rego and Batista, 2003). As a result, only successful coupling of bare TiO_2_ nanoparticles to Dopac-modified luciferase will result in the electronic interaction manifested by the appearance of the visible absorption band (Paunesku et al., 2003; Rajh et al., 2004) of the nanoconjugate (TiO_2_–Dopac–Luciferase, TiDoL). Therefore, nanocomposite coupling was monitored by the appearance of visible absorption at 550 nm (Supplementary Figures S1B,C) and the change in the zeta potential and electrophoretic mobility by laser Doppler micro-electrophoresis.

The formation of the TiDoL complex affected the intensity and, to a smaller extent, the kinetics of luciferase bioluminescence (Figures 1B,C). When the TiDoL nanoparticles were activated with ATP and luciferin, the luminescence was reduced to 60–65% compared to free luciferase, independent of the ATP concentration (Supplementary Figure S3). The decay kinetics of both quenched and unquenched samples were similar; however, somewhat faster kinetics of a sub-millisecond fraction of the luminescence decay was observed, while subsecond decay was unaffected. Interestingly, we did not observe luminescence quenching of unconjugated free luciferase in the presence of TiO_2_-Dopac nanoparticles, probably due to the low probability of interaction between free luciferase and TiO_2_-Dopac nanoparticles in diluted solutions (10 nM). This finding suggests that the quenching is a result of a strongly distance-dependent interaction, such as energy or electron transfer, and occurs only when luciferase activation is confined to the nanoparticle surface. Independent of the quenching mechanism, both energy and electron transfer results in a charge separation with holes localized on the surface modifier and electrons in TiO_2_ nanoparticles (Rajh et al., 2002). We investigated the charge separation by monitoring electron formation in TiO_2_ nanoparticles. For this purpose, we constructed an electrochemical cell with a TiDoL working electrode and Pt counter electrode (Figure 1D). We measured the current produced after injection of luciferin and ATP solutions. Immediately after the injection, we observed the rise of the current (Supplementary Figure S4), with the current intensity dependent on ATP and luciferin concentration in a dose-dependent manner (Figure 1E), echoing the dependence of TiDoL photoluminescence on ATP and luciferin concentrations (Figure 1F). The range of ATP concentrations varied from low micro-molar levels (ATP concentration in healthy tissues) to millimolar concentration (ATP concentration in tumor tissues). Once ATP concentration reached the ATP levels found in tumor tissues, we observed a significant electric current which was accompanied by a production of superoxide radicals O_2_^−^· that was detected by dihydrorhodamine (DHR) 123 fluorescence (Dimitrijevic et al., 2009). The fluorescence of DHR 123 was suppressed in the presence of a millimolar concentration of p-benzo-quinone (an O_2_^−^· scavenger), confirming that superoxide radicals are the major reactive oxygen species (ROS) formed upon luciferin activation of TiDoL. The concentration of O_2_^−^· was found to be 0.5–5 µM depending on the luciferin and ATP concentration.

### 3.2 Real-time monitoring of the interaction of TiDoL with HCT116 cells

**FIGURE 2:**
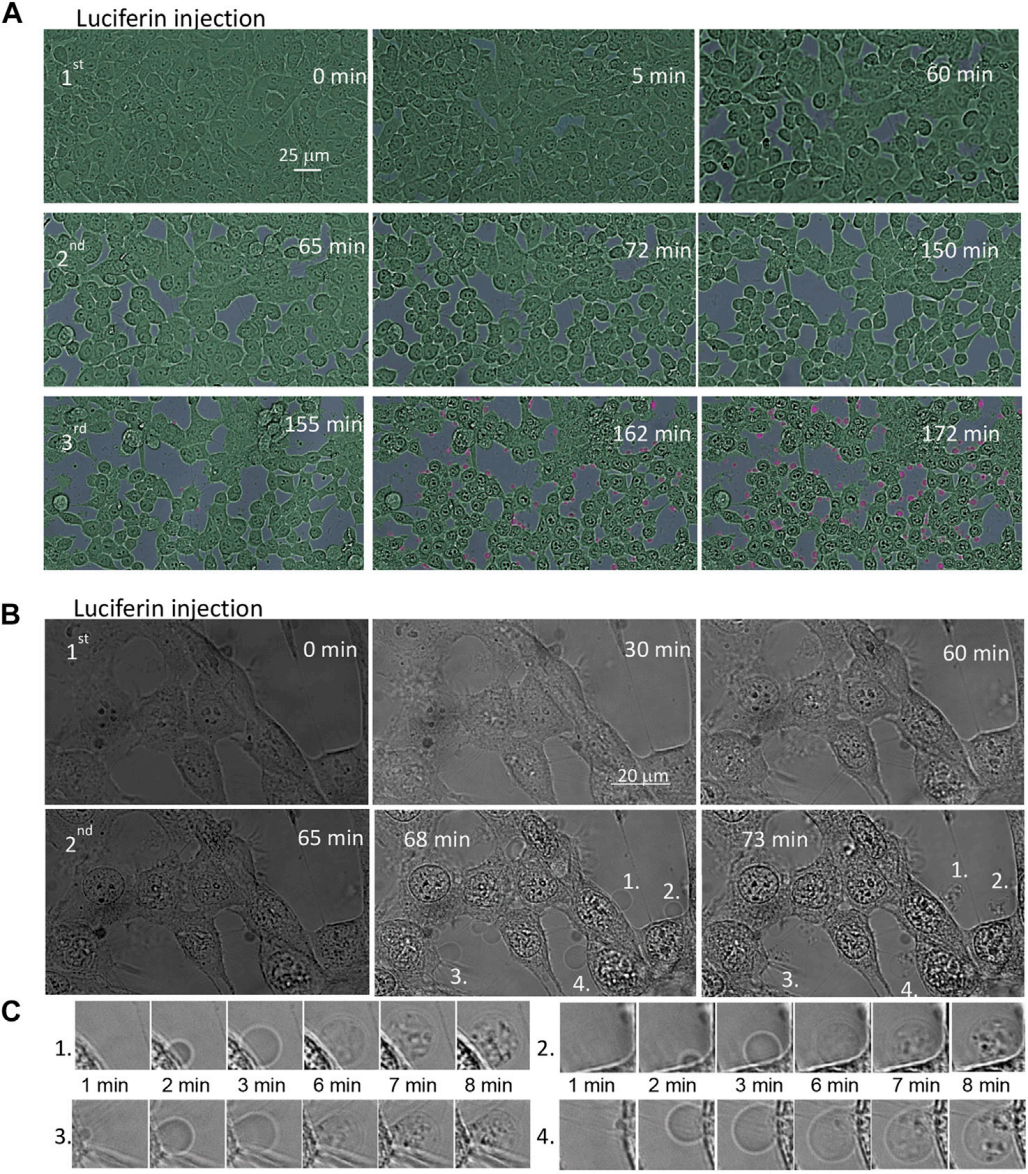
Time course of the morphological changes of the HCT116 colon cancer cell line treated with 150 nM TiDoL in the presence of ATP (20 μM). After 20 min of incubation, an aliquot of luciferin (15 μM final concentration) was injected to the well and the response of the cells was monitored using the transmission mode of a confocal microscope. Cells incubated **(A)** in the McCoy culture media (absorption ≤600 nm); three aliquots of luciferin were added (45 μM final concentration) at 60 min intervals to induce cell shrinking (cell interspace shaded in gray) and budding (shaded in pink). Magnification ×40. For clarity, cells were shaded in green and extracellular space in gray. Cells incubated **(B)** in Hanks’ Balanced Salt Solution (absorption ≤300 nm); two aliquots of luciferin were sufficient to cause cell budding. Magnification ×126. **(C)** Time course of formation, growth, and transfer of cell material to the apoptotic bodies after the second luciferin injection.

The dynamic processes of self-illuminating TiDoL nanoparticles within the cell environment *in vitro* were monitored by *in situ* confocal microscopy. This technique is particularly advantageous because it enables the direct correlation of variations in the sample morphology with the sample perturbation (same sample before and after stimuli). Figure 2A and Supplementary Movie S1 show the evolution of the cell morphology upon injection of ATP and luciferin to HCT116 colon cancer cells incubated with TiDoL in cell culture media (light absorption *λ* ≤ 600 nm) at different times after injection of luciferin. Shortly after the first injection (5 min), cell rounding, shrinking, and the appearance of an enlarged intercellular spacing are observed. Cell shrinking continues till it was 85% of the initial surface coverage 50 min after injection (15% intercellular spacing shaded in gray), and after that time, no additional changes were visible. Cell shrinking accompanied by slow detachment from the well support (manifested as a change of the focus plane) is indicative of cell death (Mills et al., 1999). Cells, however, remain with an intact plasma membrane, indicative of the typical morphology of apoptosis. Injection of the second aliquot of luciferin again accelerates changes in cell morphology. Cells shrink further to 60% of their initial surface coverage 85 min after injection of the second dose of luciferin (40% intercellular spacing shaded in gray) and blebbing of the cells is observed throughout the well (Supplementary Movie S1). Despite the decrease in volume, cells remain contained within an intact membrane and are interconnected with stretched actin across large empty intercellular spacing. It is important to note that the addition of supplemental ATP did not induce further changes in cell morphology. Injection of the third aliquot of luciferin rapidly causes even more drastic changes, and after injection, the nuclear membrane starts darkening and nuclear chromatin starts condensing, enhancing the contrast of the image. Concomitantly, cells start forming apoptotic bodies or apoptosomes. The formation of these membrane-enclosed apoptotic bodies is a critical aspect of TiDoL-induced cell death as apoptotic bodies are phagocyted and digested by nearby resident cells (Mills et al., 1999).

This sequence of events was even faster and more clearly observed when cells are exposed to TiDoL/ATP/luciferin in a buffer solution that does not attenuate 560 nm light, suggesting that the energy transfer from luciferase-activated luciferin is a primary mechanism of TiDoL activation (Figure 2B). Subsequent injection of an aliquot of luciferin leads to quick appearance (3 min) of apoptotic bodies, enhanced contrasting of the cell features, and reorganization of the cell chromatin. We observe packaging of cellular contents into membrane-enclosed apoptotic bodies (Lynch et al., 2017) (Figure 2C). These apoptotic bodies encapsulate the cell material and can be recognized, engulfed, and ingested by macrophages *in vivo,* enabling the clearance of apoptotic cells in the early stage of phagocytosis (Park et al., 2009; Akers et al., 2013). It is important to note that apoptotic processes were not limited to the imaging area. Post-treatment imaging of the cells in the distant areas that were not exposed to imaging light showed the same signs of apoptosis at near 100% efficiency (Supplementary Figure S5), confirming that the radicals formed in the entire sample as a result of the interaction of luciferin and ATP-powered TiDoL are responsible for inducing cell apoptosis.

### 3.3 *Ex situ* study of the interaction of TiDoL with HCT116 cells using staining

To further understand the process of cell death in colorectal cancerous HCT116 cells *in vitro* induced by the TiDoL/ATP/ luciferin system, we investigated their activity *ex situ* using cell staining methods. The HCT116 cells were incubated with TiDoL (or controls with concentrations used for *in situ* measurements, Supplementary Table S2 and Supplementary Figure S6) and activated by two sequential aliquots of luciferin 30 min apart in the CO_2_ incubator. Subsequently, live cells were washed and stained using fluorescent wheat germ agglutinin (WGA, labeled with green Alexa Fluor^®^ 488) and MitoTracker^®^ Red. WGA is a lectin that binds glycopeptides, and profuse binding of WGA to the membrane of HCT116 cells demonstrates strong glycosylation of their surface receptors. MitoTracker^®^ is a positively charged dye that labels mitochondria within live cells utilizing their membrane potential.

However, it is chemically reactive, linking to thiol groups in the mitochondria. The dye becomes permanently bound to the thiol-containing proteins in the mitochondria.

**FIGURE 3:**
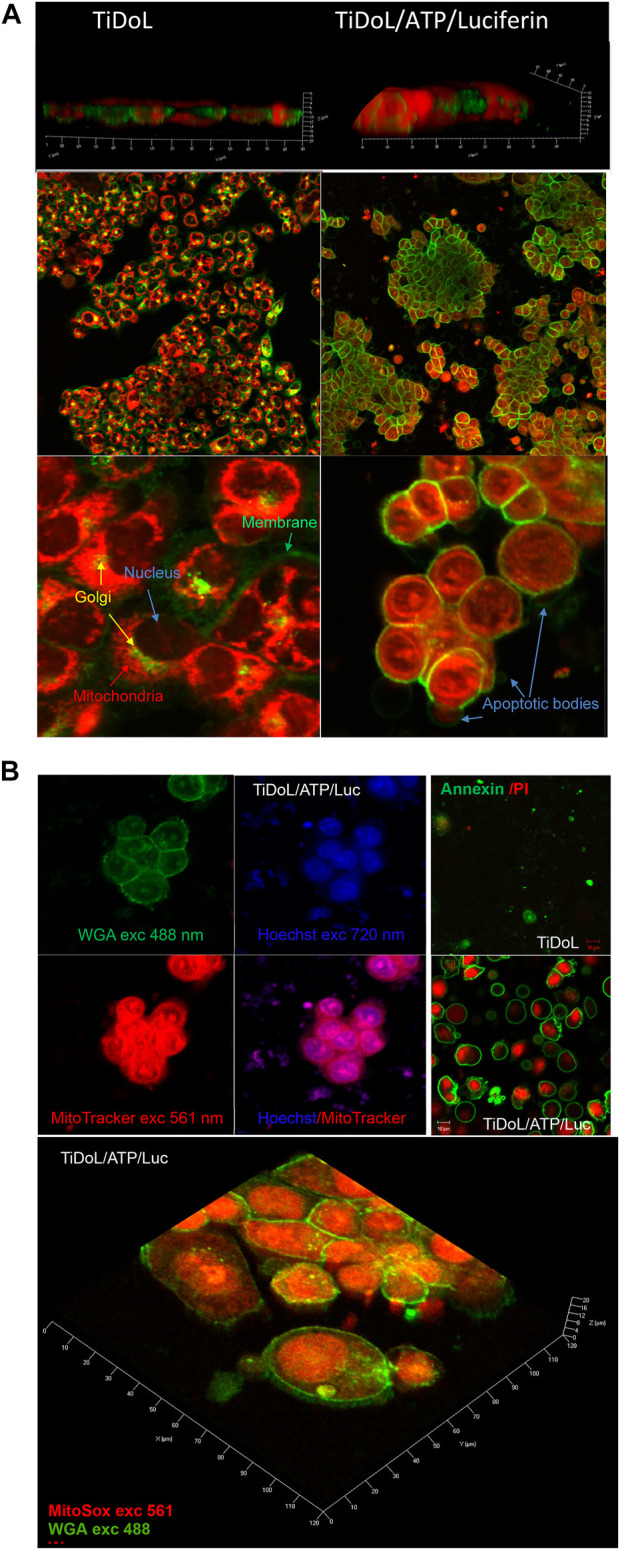
**(A)** Morphological changes in HCT116 cells upon treatment with TiDoL/ATP/luciferin observed staining of the membrane and trans-Golgi with WGA labeled with green Alexa Fluor^®^ 488 and mitochondria staining using MitoTracker Red 12 h after treatment. The left panel depicts viable cells imaged in HBSS after treatment with 150 nM TiDoL, and the right panel depicts cells treated with 150 nM TiDoL in the presence of 20 μM ATP and two aliquots of 15 μM luciferin added in the intervals of 30 min. Top panels were observed under ×20 magnification, while the bottom panels were observed under ×100 magnification. **(B)** Top left: HCT116 cells after treatment with TiDoL/ATP/luciferin observed by staining the membrane with WGA (green), thiol-containing peptides with MitoTracker (red), and ds DNA with Hoechst 33,342 (blue) 12 h after treatment. Top right: Annexin-FITC and propidium iodide staining before and after treatment with luciferin. Bottom: 3D images of treated cells stained with MitoSox Red for obtaining the distribution of superoxide radicals within treated cells. For comparison, superoxide was below the detection limit in the viable cells.

Figure 3A shows stained HCT116 cells incubated with TiDoL before (left) and after treatment with luciferin (right). Two images show significant morphological changes after luciferin treatment. All the cells incubated with TiDoL only (left) remain unchanged and have well-defined cytoplasmic membranes (green), Golgi apparatus (yellow), and polarized mitochondria (red). The 3D images also show that cells are spread and adhered to the support (Supplementary Figure S7). After treatment with luciferin, the cells lose their morphological features, shrink laterally, and thicken. However, they still retain strong and intact cytoplasmic membranes (right). The Golgi apparatus disappears, the nuclear membrane becomes permeable, and thiol-containing proteins from mitochondria labeled with MitoTracker become uniformly distributed throughout the cytoplasmic and nuclear regions. There is, however, an indication of the existence of the nuclear membrane of enlarged nuclei (Supplementary Figure S8, faint WGA circle inside the cell membrane outline). Importantly, similar to our findings by *in situ* imaging, new enclosed pressurized membrane vesicles suggestive of apoptotic bodies are also observed, with cells exchanging their content with apoptotic bodies, including thiol-containing peptides and proteins.

Further study of the apoptotic pathway was investigated by imaging the nuclei using Hoechst 33,342 stain, a dye often used to distinguish condensed pyknotic nuclei in apoptotic cells (Manchukonda et al., 2013; Rajh et al., 2014). Figure 3B shows images of the cells by laser excitation using 488 and 561 nm and two-photon excitation using 720 nm light. The image obtained using WGA and MitoTracker depicts the intact membrane and depolarized mitochondria with protein contents throughout both the cytoplasmic and nuclear regions, while two-photon imaging using Hoechst shows pyknotic nuclei, typical of apoptotic cell death. Nuclear chromatin condensation and chromosomal DNA fragmentation are well-described as key features in apoptosis (Manchukonda et al., 2013; Pawłowska et al., 2020). Chromatin condensation is microscopically visible after staining with blue-fluorescent Hoechst 33,342 dye and dense chromatin aggregates typically near the nuclear membrane are observed. Condensed chromatin is the result of a specific DNA fragmentation in the nuclei *via* cleavage by endogenous endonucleases and appears as pyknotic nuclei. The large nuclei and scant cytoplasm observed in two-photon measurements are also indicative of apoptosis (Elmore, 2007). The presence of DNA can also be observed in apoptotic bodies colocalized with thiol-containing proteins outside the cell compartment. MitoSox red staining also indicates enhanced oxidative stress (superoxide was below the detection limit in the viable cells) (Oropesa-Avila et al., 2014). High fluorescence of oxidized MitoSox dye was detected in the nuclear region of TiDoL/luciferin-treated cells and in apoptotic bodies which can be attributed to the higher intensity of the Mitosox red fluorescence when bound to double-stranded DNA rather than to the higher concentrations of superoxide radicals in these cell regions. The detected concentration of O_2_^−^· in these regions was 60% larger than the one in non-treated control cells. Considering that O_2_^−^· steady-state concentrations are estimated to be on the nanomolar level in control cells (Radi, 2018), the O_2_^−^·levels in TiDoL-luciferin-treated cells exceed µM levels.

The apoptotic mechanism of cell death was also confirmed using Annexin/propidium iodide apoptosis assay (Figure 3B). Annexin V is a cell protein that binds phosphatidylserines, membrane phospholipids that are held on the inner layer of the cell membrane, and Annexin V does not attach to viable cells. During the early stage of apoptosis, the phosphatidylserines become exposed on the outer layer of the cell membrane, and Annexin V labels phosphatidylserine sites on the membrane surface. At this stage, the cell membrane remains intact and propidium iodide cannot enter to cell interior. At later stages of apoptosis, the membrane becomes somewhat permeable and in addition to annexin binding (green luminescence) propidium iodide penetrates the cell interior and shows red luminescence of the cell interior. In ATP and luciferin-treated cells containing TiDoL, we observe cells in both early and later stages of apoptosis and pressurized apoptotic bodies with phosphatidylserines exposed on their outer membrane surface (green circles). Some of the apoptotic bodies detach from the treated cells and become apo-extracellular vesicles with phosphatidylserines exposed on their outer membrane surface.

Morphological changes confirm the apoptotic pathway of cell death upon ATP initiated *in vitro* treatment of the cancer cells using TiDoL nanoparticles. This is a critical finding, as programmed cell death is characterized by the degradation of cell components within apoptotic cells while their plasma membrane remains intact. It is reported that apoptosis is a physiologically advantageous way of cell death because apoptotic cells can be removed by phagocytosis and digested by nearby resident cells before they lose their outer permeability barrier, thus preventing the induction of inflammatory responses to dying cells and potentially harmful secondary effects (Oropesa-Avila et al., 2014).

### 3.4 Functionalization of TiDoL with anti-EGFR antibody C225

**FIGURE 4:**
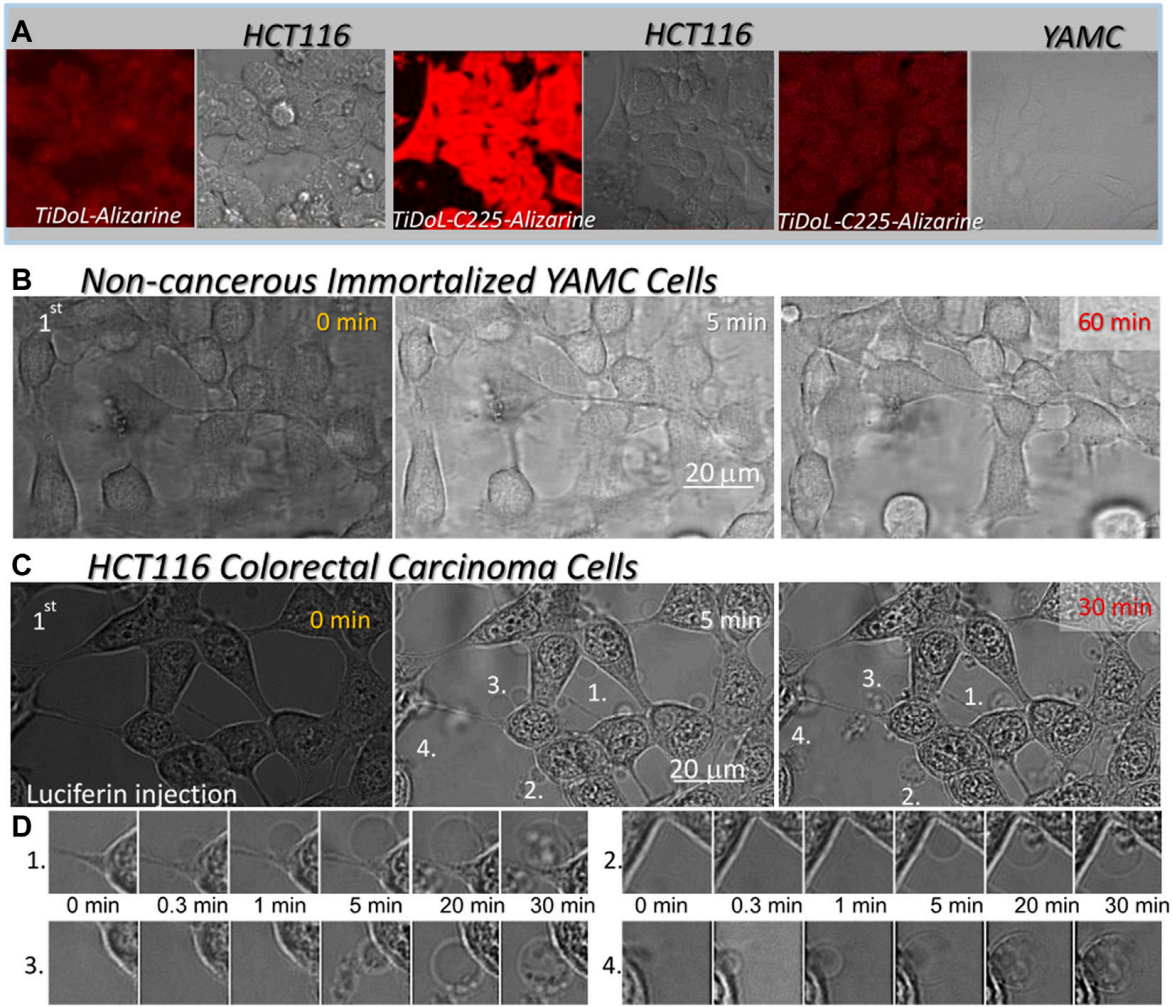
**(A)** Adsorption of nanoconjugates w/o the C225 antibody on HCT116 cells and EGFR(-) immortalized young adult mouse colon (YAMC) cells. Nanocomposites are visualized by imaging fluorescence of alizarin bound to TiO_2_ nanoparticles (red) using laser excitation 560 nm and cells (gray) are imaged in the transmission mode. Magnification ×40. **(B)** Time course of the morphological changes of the YAMC colon cell line treated with 150 nM TiDoL-C225. Magnification ×126. **(C)** Time course of the morphological changes of the HCT116 colon cancer cell line treated with 150 nM TiDoL-C225All conditions are the same as in Figure 2B and budding of the cells starts 30 s after first luciferin injections. Magnification ×126. **(D)** Time course of formation, growth, and transfer of cell material to the apoptotic bodies after the first luciferin injection.

Selectivity of TiDoL nanoparticles toward cancer cells and their efficiency toward programmed cell death were further enhanced by the functionalization of TiDoL nanoparticles with monoclonal antibody C225 that increases retention of the nanocomposites in tumor regions (Scott et al., 2012). C225 (or Cetuximab^®^) is a chimeric monoclonal antibody directed against the epidermal growth factor (EGFR) and binds to the extracellular domain of the EGFR. It prevents dimerization of the receptor, resulting in anti-proliferative effects and hinders EGFR-dependent primary tumor growth and metastasis (Liao and Carpenter, 2009). The EGFR is overexpressed on the cell membranes of various solid tumors and is highly expressed on the surface of the HCT116 cell line (EGFR+). C225 have been used successfully as a therapeutic agent in treating metastatic colorectal and head and neck cancers by targeting specific EGFR + cells (Scott et al., 2012). Combining this antibody therapy with photocatalytic nanoparticles can induce correlated activities of photoinduced and antibody therapies of photocatalytic nanoparticles, enhancing the rate and efficiency of targeted therapy (Xu et al., 2007; Rozhkova et al., 2009; Elvira et al., 2012). To visualize antibody-facilitated adsorption (retention) of TiDoL-C225 composites within the cells that express EGFR, we modified nanoconjugates with alizarin, an enediol dye that changes the absorption and fluorescence of TiO2 nanoparticles (Supplementary Figure S1) (Rajh et al., 2002). Figure 4A clearly shows enhanced retention of TiDoL-C225 nanocomposites on the surface of EGFR + colorectal cancerous HCT116 cells (center) while their retention (adsorption) in conditionally immortalized young adult mouse colonocyte (YAMC) cells that do not express EGFR (EGFR(−)) is negligible under the same conditions (right). Likewise, retention of TiDoL nanoconjugates in the absence of the C225 antibody on HC116 cells (EGFR(+)) was negligible as shown by the faint red fluorescence of alizarin-TiDoL (left). Similar poor adherence of TiDoL-C225 was observed for EGFR(−) non-transformed fibroblast CT26 cells (Supplementary Figure S11). These results suggest weak retention and poor activation of TiDoL-C225 within the healthy tissue compared to the one when TiDoL-C225 is integrated with cancerous EGFR(+) cells. Indeed, Figure 4B shows the time sequence of morphological changes associated with the activation of TiDoL-C225 within immortalized YAMC cells (EGFR(−)) that show minor changes even 60 min after activation with luciferin. The composite does not induce morphological changes in YAMC cells even after 3 doses of luciferin. Similar results were obtained for CT26 EGFR-non-transformed fibroblast cells (Supplementary Figures S10).

Indeed, the activation of TiDoL-C225 within HCT116 cells (EGFR+) resulted in the rapid development of the signs of the final stages of apoptotic death (Figure 4C; Supplementary Movie S2). Cell budding was accompanied by darkening of the nuclear membrane and enhancement of the image contrast as a result of chromatin rearrangement. Due to this rapid budding, the cells do not have time to go through the significant shrinking phase; however, they still show strong and intact cytoplasmic membranes that extend to apoptotic bodies (Figure 4D). Cells start to transfer their content to the apoptotic bodies much later compared to the same process in treatment with TiDoL conjugate in the absence of C225 (Figure 2C). Importantly, TiDoL-C225 nanocomposites induced cell death at a concentration as low as 5 nM (0.5 μg/ml TiO_2_) compared to 20 nM TiDoL under the same conditions. Antibodies provide specificity to the nanoparticles, increasing their retention within targeted cells that express cognate antigens on the membrane surface. Hence, composites are activated in close proximity to biological targets of interest, enhancing the efficiency of nanocomposites during therapy.

### 3.5 *In vivo* study of the interaction of TiDoL and TiDoL-C225 with tumors

**FIGURE 5:**
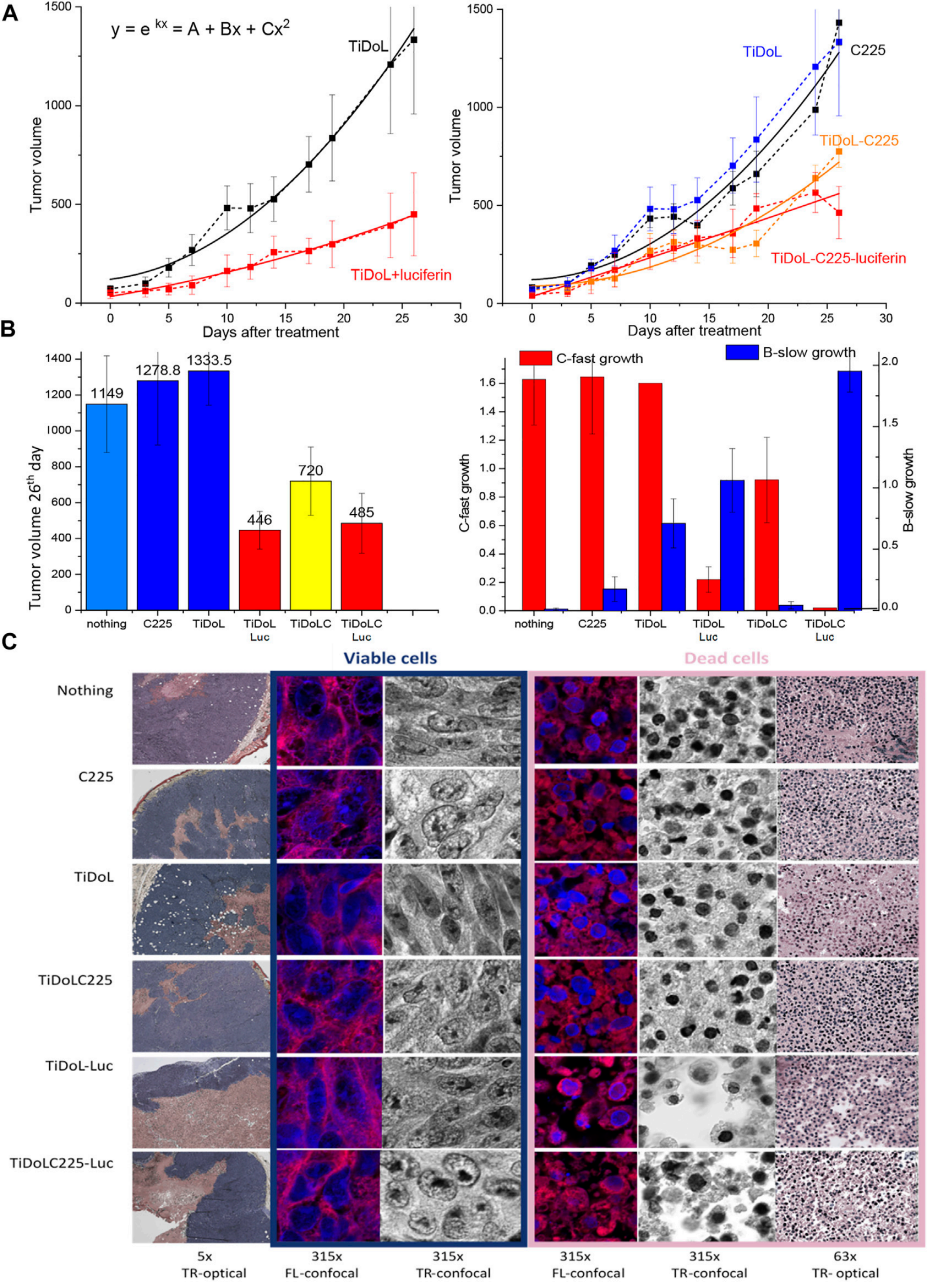
*In vivo* study of tumor growth in nude mice (HCT116 xenografts). **(A)** Evolution of tumors treated with TiDoL (left) and TiDoLC225 (right) w/o activation with luciferin. Each point is an average of six measurements. Statistical significance was determined using Student’s *t-*test as *p* < 0.0068 for TiDoL and *p* < 0.0083 for TiDoL-C225. Data are fitted with quadratic polynomial dependence as an approximation of exponential growth. **(B)** Color-coded histogram of tumor sizes 26 days after the treatment of nude mice in conjunction with the histogram of the linear (blue) and quadratic (red) coefficient of tumor growth obtained from data fitting. **(C)** Transmission optical (TR-optical) and laser confocal fluorescence (FL-confocal) and optical (TR-confocal) images of H&E-stained tumor slices obtained by sectioning the tumors 26 days after the treatment at different magnifications.

To demonstrate the applicability of our approach *in vivo,* we administrated a single dose of 50 μl TiDoL (6 μM), TiDoL-C225 (4 μM), and appropriate controls into nude mice bearing the aggressive HCT116 xenograft tumor model. Luciferin was administrated intraperitoneally, one dose 1 h and the other 48 h after TiDoL treatment. We observed significant differences in cancer growth between the tumors with and without luciferin administration in TiDoL-treated nude mice (Figure 5). While those tumors that did not receive luciferin continued exponential growth (Elmouelhi et al., 2008), the progress of luciferin-treated tumors slows down, and 26 days after the treatment, becomes three times smaller on average compared to those that did not receive luciferin. Each point shown in Figure 5A, left is an average of six tumor measurements, and their statistical significance was determined using Student’s *t*-test. For the data obtained for the 26th day after TiDoL injection, statistical significance was *p* = 0.0068. Cell growth of all tumors was fitted with a polynomial (parabolic) function and it was found that cell growth in control tumors is dominated by a fast quadratic term, while those administered with luciferin and nanoconjugates show slow linear growth. These findings suggest that upon activation of TiDoL in the tumors with luciferin, a process that competes with cell growth is initiated, limiting the exponential proliferation rate of the tumor.

Administration of targeted TiDoL nanoconjugates modified with the C225 antibody also resulted in limiting the growth of the tumor when treated with luciferin. It was observed that 26 days after treatment, the tumors show three times smaller size on average compared to those treated with antibody C225 only. Each point shown in Figure 5B is an average of six tumor measurements and their statistical significance for data obtained 26 days after injection was *p* = 0.0083. The treatment of the tumor with TiDoL-C225 nanoconjugates only, without administration of luciferin, also slows down the growth rate of the tumor on average in agreement with a previously observed enhanced toxicity of C225 when conjugated to nanoparticles (Qian et al., 2014). However, the addition of luciferin further slows down the growth rate mechanism fostering linear growth of tumors, and luciferin-treated tumors grow to 30% of the size measured in the controls (Figure 5A, right). Histologic analysis of non-activated control H&E-stained tumors 26 days after the treatment shows that tumors have large areas of growing viable cells and smaller necrosis areas confined to the center of the tumor (Figure 5C), typically attributed to fast-growing tumors outgrowing their blood supply (Lotze and Demarco, 2004). H&E-stained tumors treated by TiDoL and TiDoL-C225 activated with luciferin, however, show large dead cells and denuded areas. Large, denuded areas in TiDoL-C225/luciferin-activated tumors suggest macrophage-assisted clearance of dead cells. These areas are surrounded by a thin belt of viable growing cancerous cells. High magnification (×315) shows faint hematoxylin-stained DNA/RNA in growing cells distributed throughout the nuclei and condensed in a highly stained nucleolus. Dead tumor areas, however, show much smaller pyknotic nuclei in condensed cells. These cells exhibit large denuded areas that in the case of TiDoL-treated tumors are very clean, while in TiDoL-C225-treated tumors show residual debris adjacent to denuded tumor areas. Some of the debris contains DNA/RNA, while others contain mainly proteins stained by eosin. These results suggest that while large tumors in the control group continue to grow, the tumors treated and activated with luciferin show reduced tumor size with cancer cell removal competing with new tumor growth, suggesting the need for tuning of nanoconjugate pharmacology for obtaining optimal efficiency.

## 4 Conclusion

To summarize, we have developed a composite that takes advantage of the high ATP levels in a tumor microenvironment and uses photodynamic therapy for cancer treatment without using an external light source. The self-illuminating nanoparticle technology enhances existing antibody platforms to an on-demand targeted therapy confined to the regions of tumor activity. The employment of ATP-powered self-illuminating nanoparticles provides a new universal approach that uses the product of disease to target diseased tissues solely. We show that, although not optimized pharmacologically, this technology kills close to 100% of cancerous cells *in vitro* and shows a significant decrease in the tumor size *in vivo* to 30% of their original size in experimental colon cancer models. Herein, we deployed this technology against a colon cancer cell line; however, our additional work shows that the same approach is valid for a very different A172 glioblastoma brain tumor cell line when an anti-IL13 was used as an anchoring antibody instead of C225. This method minimizes the probability of the off-target release of radical species in a double lock-in approach (Augulis and Zigmantas, 2011) and enhances the potency of cell-locked *in situ* nanoparticle delivery of reactive species produced in close proximity to biological targets.

## Data Availability

The original contributions presented in the study are included in the article/Supplementary Material; further inquiries can be directed to the corresponding author.

## Supplementary material

Supplementary Movie S1 TiDoL within HCT116 cell culture.

Supplementary Movie S2 TiDoLC225 within HCT116 cell culture.

Supplementary Video S1 Self-illuminating TiDoL nanocomposites within HCT116 colorectal carcinoma cells. Video spanning 30 min before and 180 min after activation of TiDoL with luciferin (three aliquots) injected into the TiDoL–McCoy media solution, depicting morphological changes of HCT116 cells upon activation of TiDoL. Morphological changes were recorded 60 min after each luciferin injection, one frame every 20 s. Cell shrinking, detachment from the support, and cell blebbing are observed after the first luciferin injection. Injection of the second aliquot of luciferin again accelerates changes in cell morphology. Cells shrink to 60% of their initial surface coverage 85 min after injection of the second dose of luciferin. Blebbing of the cells is observed throughout the well (Supplementary Video S1). Despite the decrease in the cell volume, cells remain contained within an intact membrane and are interconnected with stretched actin across large empty intercellular spacing. The movie also shows that the addition of supplemental ATP did not induce further changes in the cell morphology. Injection of the third aliquot rapidly causes even more drastic changes, and 7 min after injection, cells start forming apoptotic bodies with concomitant reorganization and condensing of nuclear chromatin, enhancing the contrast of the image. Magnification ×40. The last section of the video depicts magnified cell budding (×126) of the cells in HBSS after the second luciferin injection.

Supplementary Video S2 Self-illuminating TiDoL linked to Cetuximab (TiDoL-C225) within colorectal carcinoma HCT116 cells and conditionally immortalized young adult mouse coloncytes (YAMC). HCT116: Video spanning 30 min before and 180 min after activation of TiDoL-C225 with luciferin (three aliquots) injected into the TiDoL-C225-HBSS solution, depicting morphological changes of HCT116 cells upon activation of TiDoL-C225 immobilized on the cell surface (Figure 4C). Morphological changes were recorded 60 min after each luciferin injection, one frame every 20 s. As soon as 20 s after luciferin injection, the cells show the final stages of apoptotic death. Cell budding is accompanied by the darkening of the nuclear membrane and enhancement of the image contrast as a result of chromatin rearrangement. Due to this rapid budding, the cells do not have time to go through the significant shrinking phase; however, they still show strong and intact cytoplasmic membranes that extend to apoptotic bodies. Cells start transferring their content into the apoptotic bodies 20 min after cell budding, which is a much longer timeframe compared to the same process in treatment with TiDoL conjugate in the absence of C225 (that occurred 5 min after cell budding, Supplementary Video S1). This difference indicates the decoupling of signaling cascades involved in the budding and transferring of cell material to the apoptotic bodies. Magnification ×126. YAMC: Video spanning 30 min before and 180 min after activation of TiDoL-C225 with luciferin (three aliquots) injected into the TiDoL-C225-HBSS solution, depicting morphological changes of YAMC cells upon activation of TiDoL-C225. TiDoL-C225 is only nonspecifically weakly adsorbed on the YAMC cell surface (Figure 4B) as they do not express EGFR. Morphological changes were recorded 60 min after each luciferin injection, one frame every 20 s. No significant changes were observed upon all three luciferin injections. Magnification ×126.
