# Supplementary material for: Triggering cell death in cancers using self-illuminating nanocomposites
Source: Front Chem. 2022 Sep 15;10:962161. doi: 10.3389/fchem.2022.962161 (PMC9521829; doi:10.3389/fchem.2022.962161)
Supplement: Supplementary file 3 [file DataSheet1.docx]

**Supplemental Information**

**Triggering Apoptotic Signaling Cascade with Self-Illuminating Nanocomposites: Exploiting the Overproduction of ATP in Cancerous Cells**

Tamara Koritarov,^1^ Benjamin Blaiszik,^1^ Syeda Fatima Z. Rizvi,^1^ Vani Konda,^2^ Vesta Valuckaite,^2^ Marc Bissonnette,^2^ Tijana Rajh^1^*

^1^Center for Nanoscale Materials, Argonne National Laboratory, 9700 S Cass Ave, Argonne IL 60540; ^2^Department of Medicine, The University of Chicago Medicine, 5841 S. Maryland Avenue, MC 4076, Chicago, IL 60637

**Table S1**. Size and surface charge of TiO_2_ nanoparticles before and after surface modification with Dopac and luciferase

| **Sample** | **Buffer** | **pH** | **Size (vol-avg) [nm]** | **Zeta Potential [mV]** | **Mobility [µmcm/Vs]** | **Conductivity [mS/cm]** |
| --- | --- | --- | --- | --- | --- | --- |
| **TiO2-Plain** | water | < 4 | 7.9 | 39.43 | 3.09 | 0.57 |
| **TiO2-Plain** | water | 7.8 | 7.5 | -7.0 | -1.10 | 0.43 |
| **TiO2-DOPAC** | PB | 7.0 | 12.7 | -27.73 | -2.17 | 9.15 |
| **Luc-PB** | PB | 7.0 | 6.2 | -5.82 | -0.46 | 8.61 |
| **TiO2-Dopac + free Luciferase (not conjugated; no EDC)** | PB | 7.0 | 39.4 | -5.34 | -0.42 | 9.61 |
| **Luciferase-Dopac + TiO2-Tiron** | PB | 7.0 | 44.8 | -7.77 | -0.61 | 10.77 |
| **TiDoL** | PB | 7.0 | 13.1 | -6.83 | -0.54 | 12.37 |

**b**

**5 nm**

**a**

**c**

0.4

**Figure S1**. HRTEM image of 5 nm TiO_2_ nanoparticles used in this work (a). Absorption spectra of 10 μM (particle concentration) TiO_2_ modified with different Dopac stoichiometry (1:1 equimolar; 1:3 corresponds to 3 molecules of Dopac per particle; 1:6 corresponds to 6 molecules per particle, etc.; This graph was used as calibration for binding of TiO_2_ particles to Dopac modified luciferase). The graph also shows emission spectrum of luciferase to illustrate the overlap of the luciferase emission with the absorption spectrum of various TiO_2_ samples (b). Absorption spectra of TiO2 nanocomposites bound to luciferase and C225 antibody in the presence of excess ligands used to enhance absorption properties of composites (c).


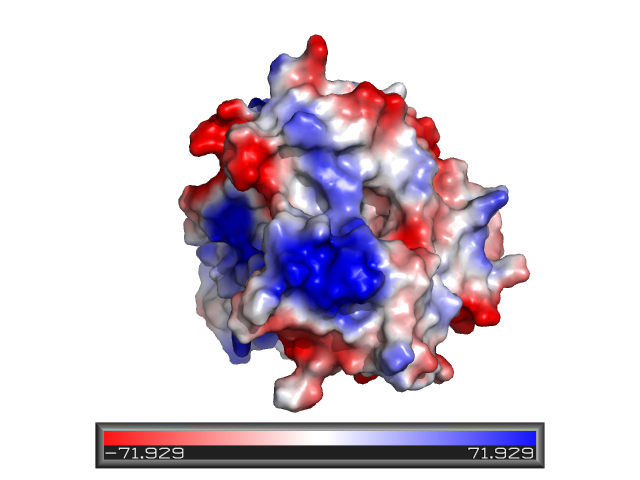

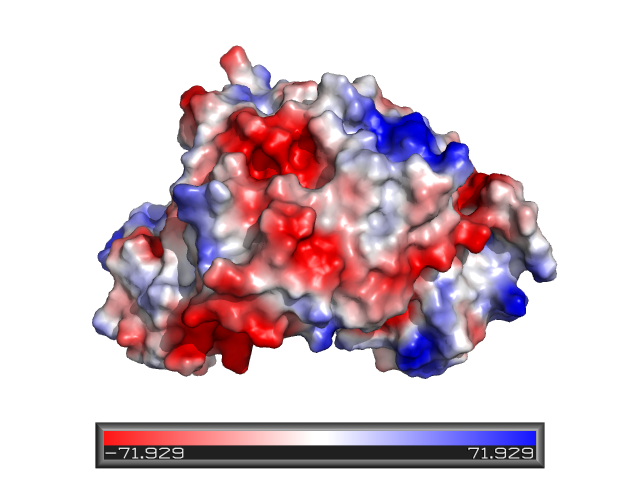


2 Arg

2 Lys

2 Arg

1 Lys

Side 1 Side 2


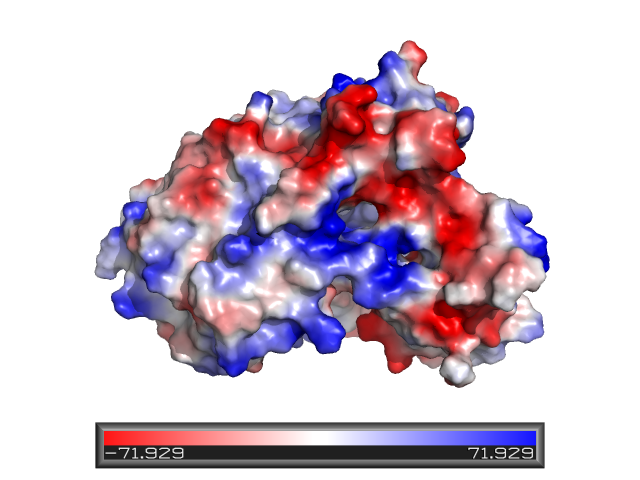

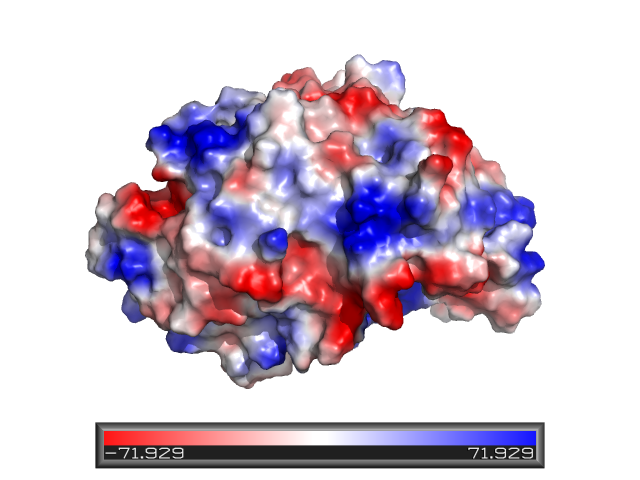


1 Arg

exposed

3 Lys

2 Arg

3 Lys

Side 3 Top

**Figure S2**. Electrostatic distribution in luciferase. Red represents negative charge (carboxyl residues) and blue positive charge (amino group residues)

|  | integrated areas (counts) | |  | |  | |  | |  |
| --- | --- | --- | --- | --- | --- | --- | --- | --- | --- |
|  | **with TiO2/DOPAC** | **no TiO2/DOPAC** | | **Quenching ratio** | | **Average** | | **peak maximum** | |
| 5mM_a | 79433 | 208218 | 0.618510407 | | 0.61056785 | | 566 nm | |  |
| 5mM_b | 73684 | 185427 | 0.602625292 | |  | | 566 nm | |  |
| 1mM_a | 60951 | 161849 | 0.623408239 | | 0.62344531 | | 566 nm | |  |
| 1mM_b | 60939 | 161849 | 0.623482382 | |  | | 566 nm | |  |
| 100uM_a | 63294 | 168015 | 0.623283635 | | 0.643527914 | | 566 nm | |  |
| 100uM_b | 54751 | 162839 | 0.663772192 | |  | | 566 nm | |  |
| 10uM_a | 10472 | 32321 | 0.676000124 | | 0.618861046 | | 566 nm | |  |
| 10uM_b | 9682 | 22091 | 0.561721968 | |  | | 566 nm | |  |
| 1uM_a | 1167 | 4105 | 0.715712546 | |  | | 566 nm | |  |

**Figure S3.** Luciferase mediated luciferin fluorescence quenching in TiDoL conjugates in the presence of different concentrations of ATP (5 mM; 1 mM; 100 μM; 10 μM and 1 μM) in conjunction with the table showing quenching ratio of fluorescence with and without presence of ToDoL. In *Taking into account Poisson distribution, which predicts that in the process of conjugation of equimolar concentrations of luciferase and TiO2 nanoparticles, 36% of TiO2 nanoparticles are not conjugated to luciferase, 60% of luciferase mediated luciferin light emission quenching is expected.*

**
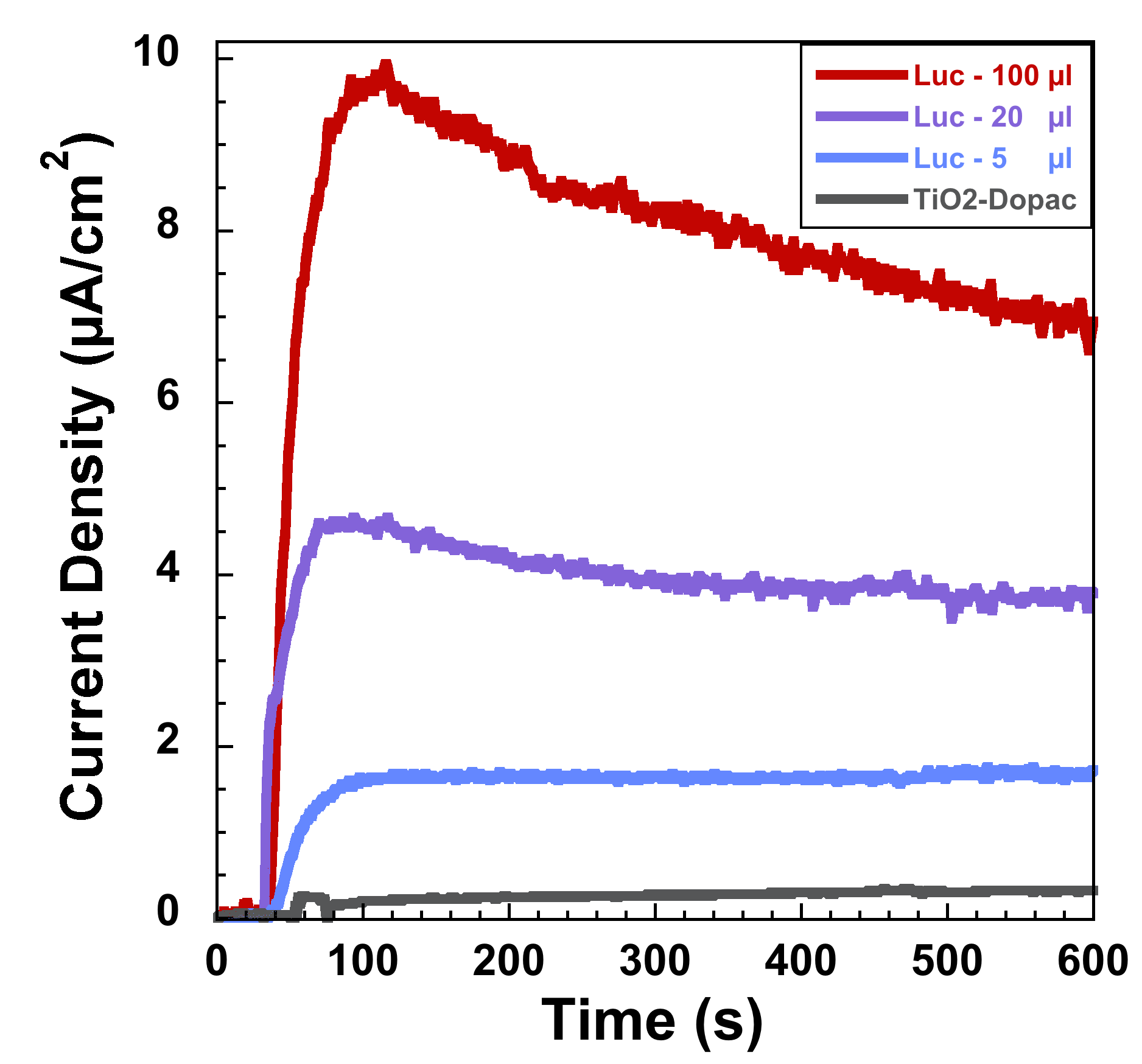

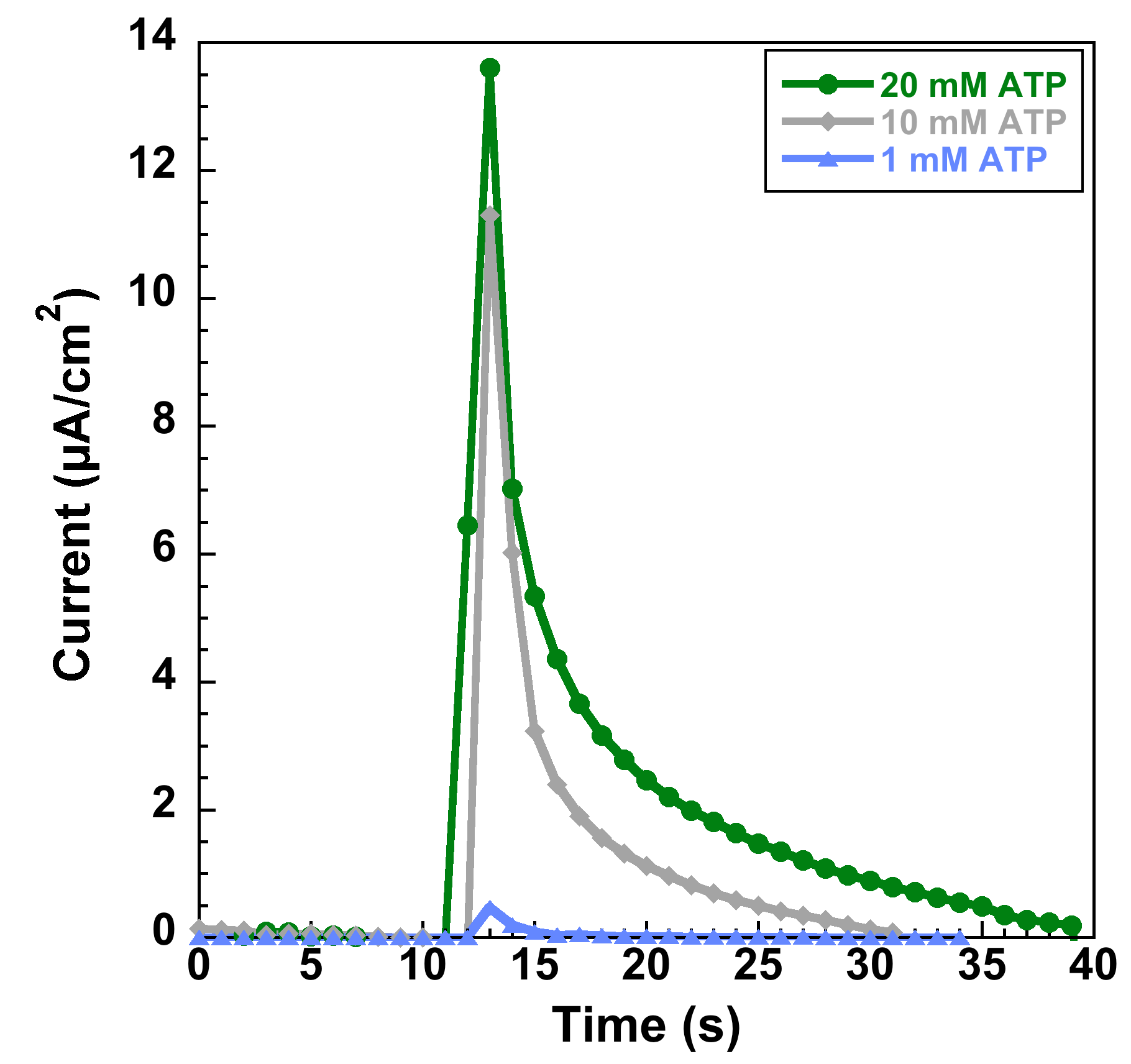
**

**Figure S4.** Time dependence of the electrical current after injection of luciferin with different concentrations of luciferin (left) and ATP (right).


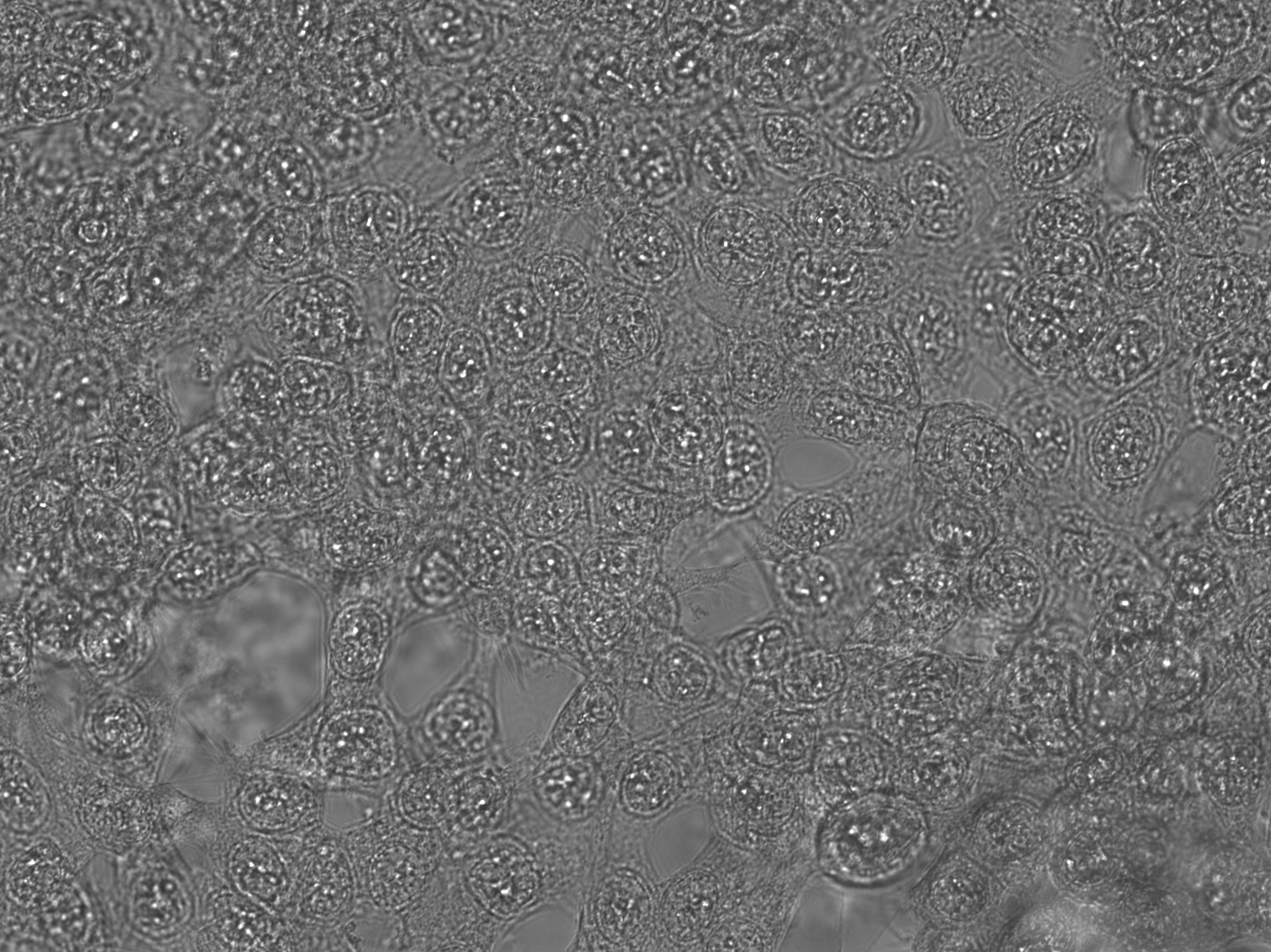


**Figure S5.** Photograph of the section of cell culture sample shown in Figure 2b that was never exposed to imaging light taken 2 hours after luciferin injection in the presence of TiDoL and ATP.

**Table S2**. TiDoL Experiment Design. Diagram of the 8-well plate of HCT116 cells and how they were treated.

| **1.**  202 uL HBSS  98 uL PBS | **2.**  202 uL HBSS  22 uL PBS  8 uL Luciferase  8 uL ATP  60 uL Luciferin (30+30) | **3.**  202 uL HBSS  30 uL NP(TiO2-DC)  8 uL Luciferase  8 uL ATP  60 uL Luciferin (30+30) | **4.**  202 uL HBSS  68 uL PBS  30 uL NP (TiDoL) |
| --- | --- | --- | --- |
| **5.**  202 uL HBSS  30 uL NP (TiDoL)  8 uL ATP  60 uL Luciferin (30+30) | **6.**  202 uL HBSS  30 uL NP (TiDoL – partial conjugation)  8 uL ATP  60 uL Luciferin (30+30) | **7.**  202 uL HBSS  30 uL PBS  8 uL ATP  60 uL Luciferin (30+30) | **8.**  202 uL HBSS  68 uL PBS  30 uL NP (TiDoL – partial conjugation) |


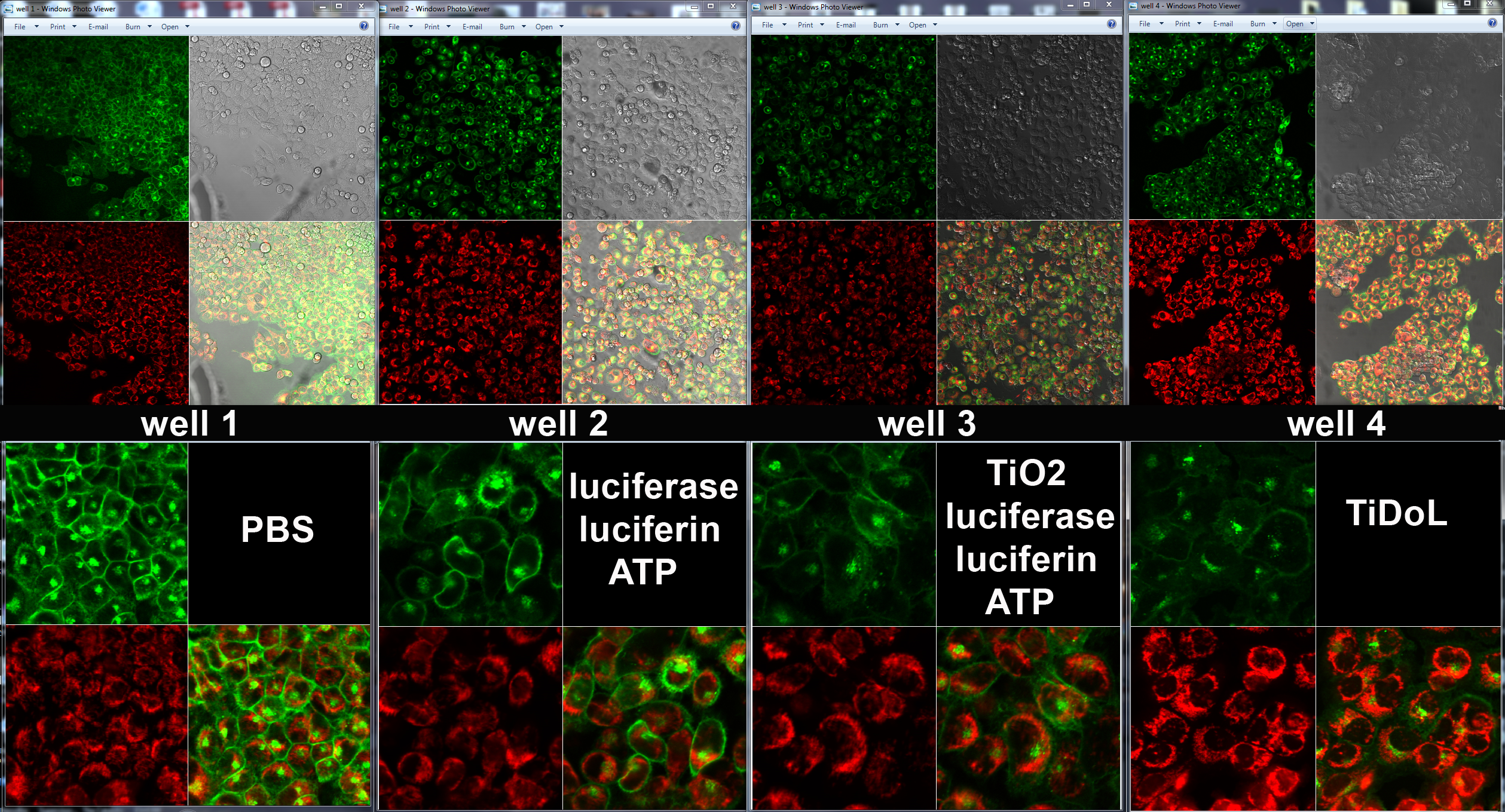


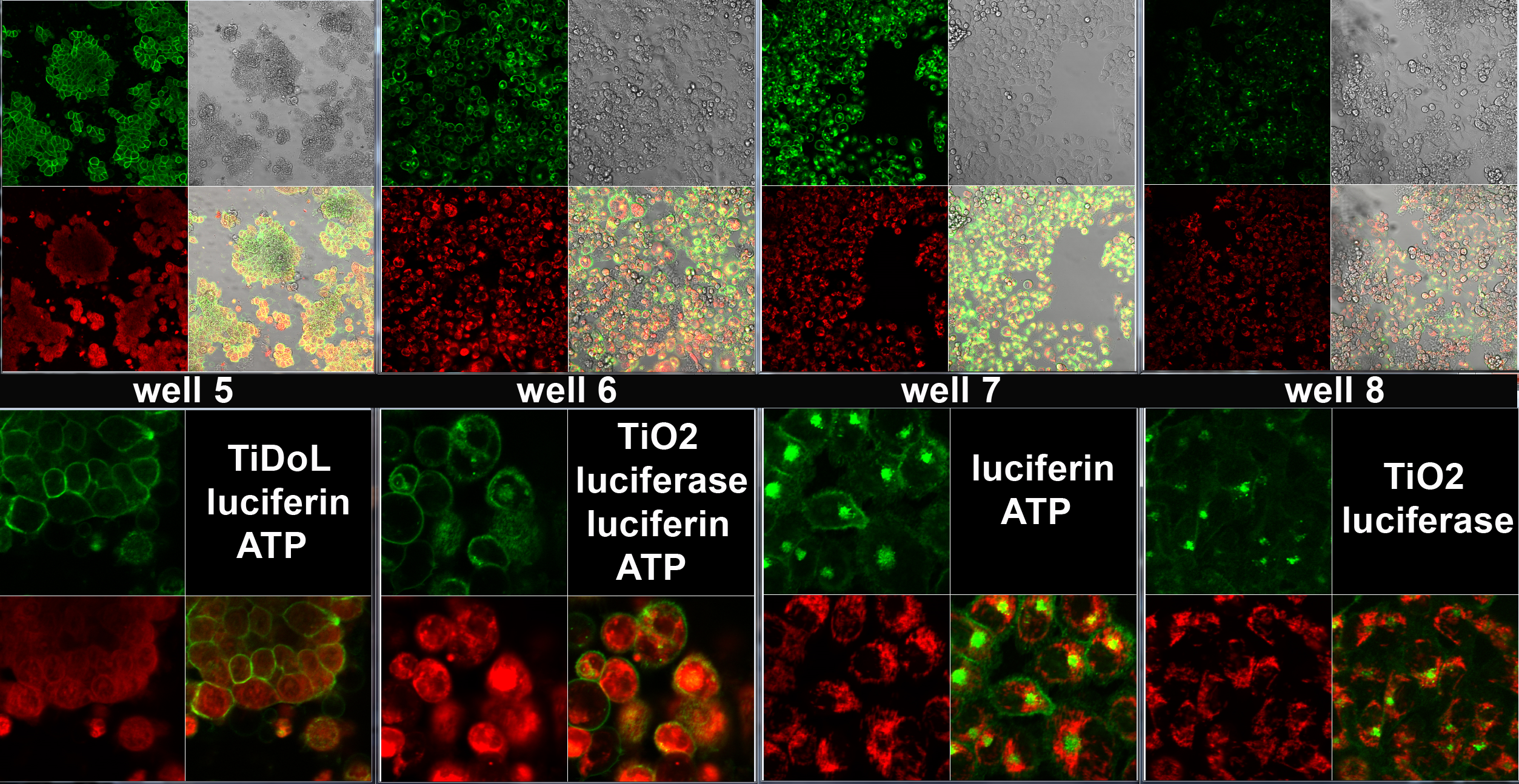


**Figure S6**. Images of 8-well plate of HCT116 cells schematically shown in Table S2. Top row 20x magnification, bottom row 100x magnification: green WGA staining, red Mitotracker red, gray transmission image, and multicolor merged overlay.


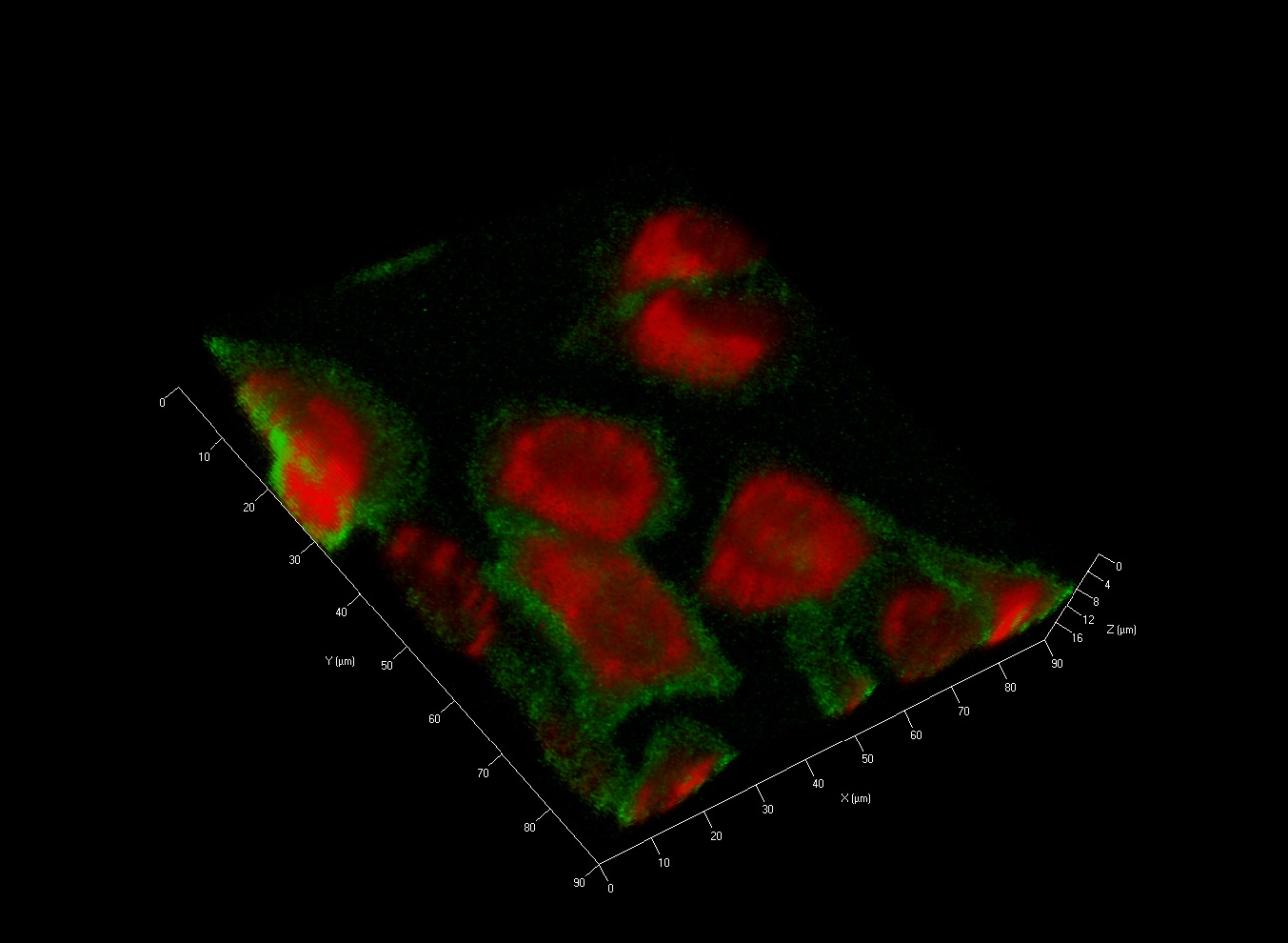

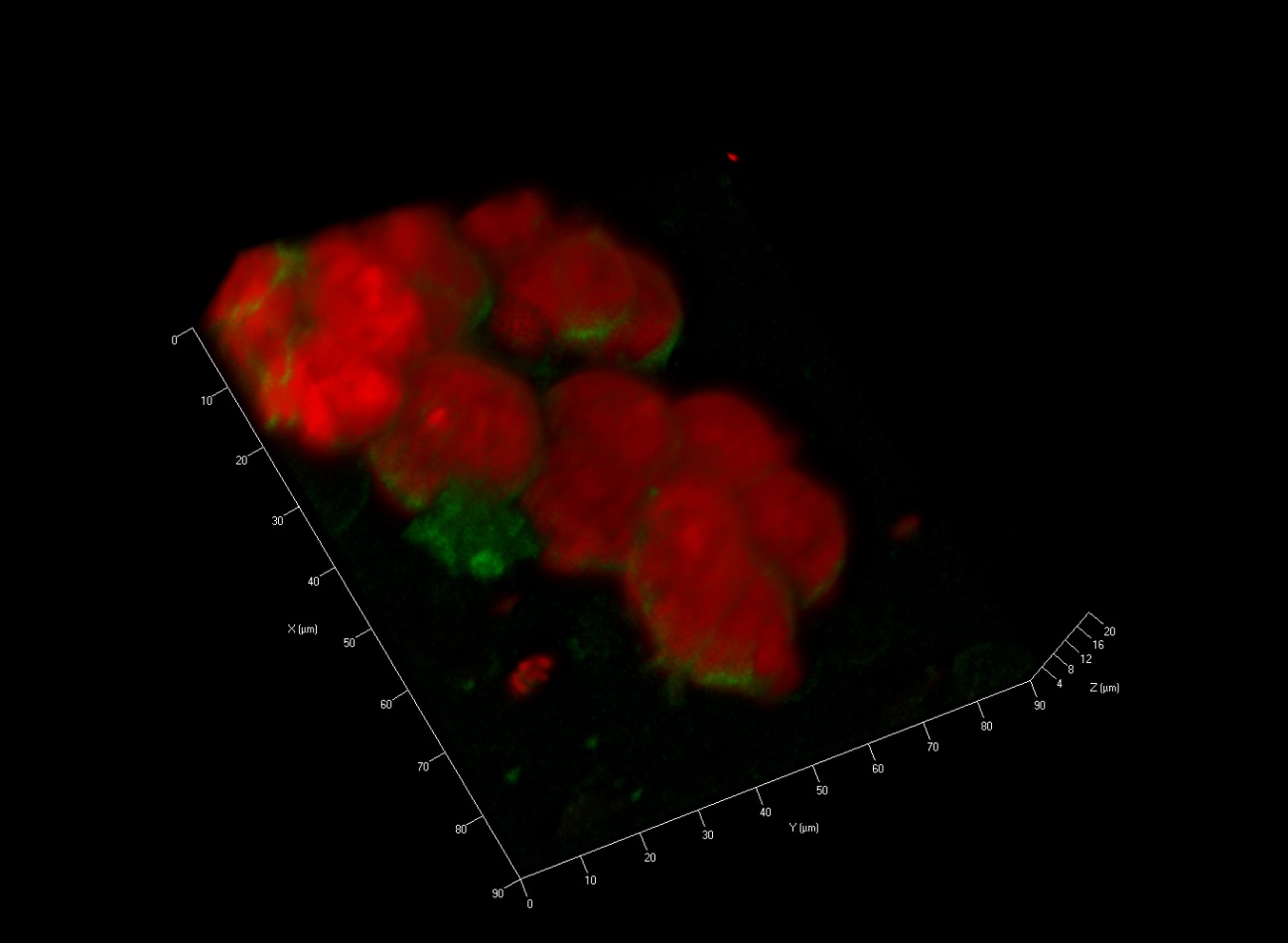


TiDoL treated luciferin activated (well 5)

TiDoL treated (well 4)

**Figure S7.** 3D images of HCT116 cells treated with TiDoL (left) and those treated with TiDoL and activated by luciferin (right).


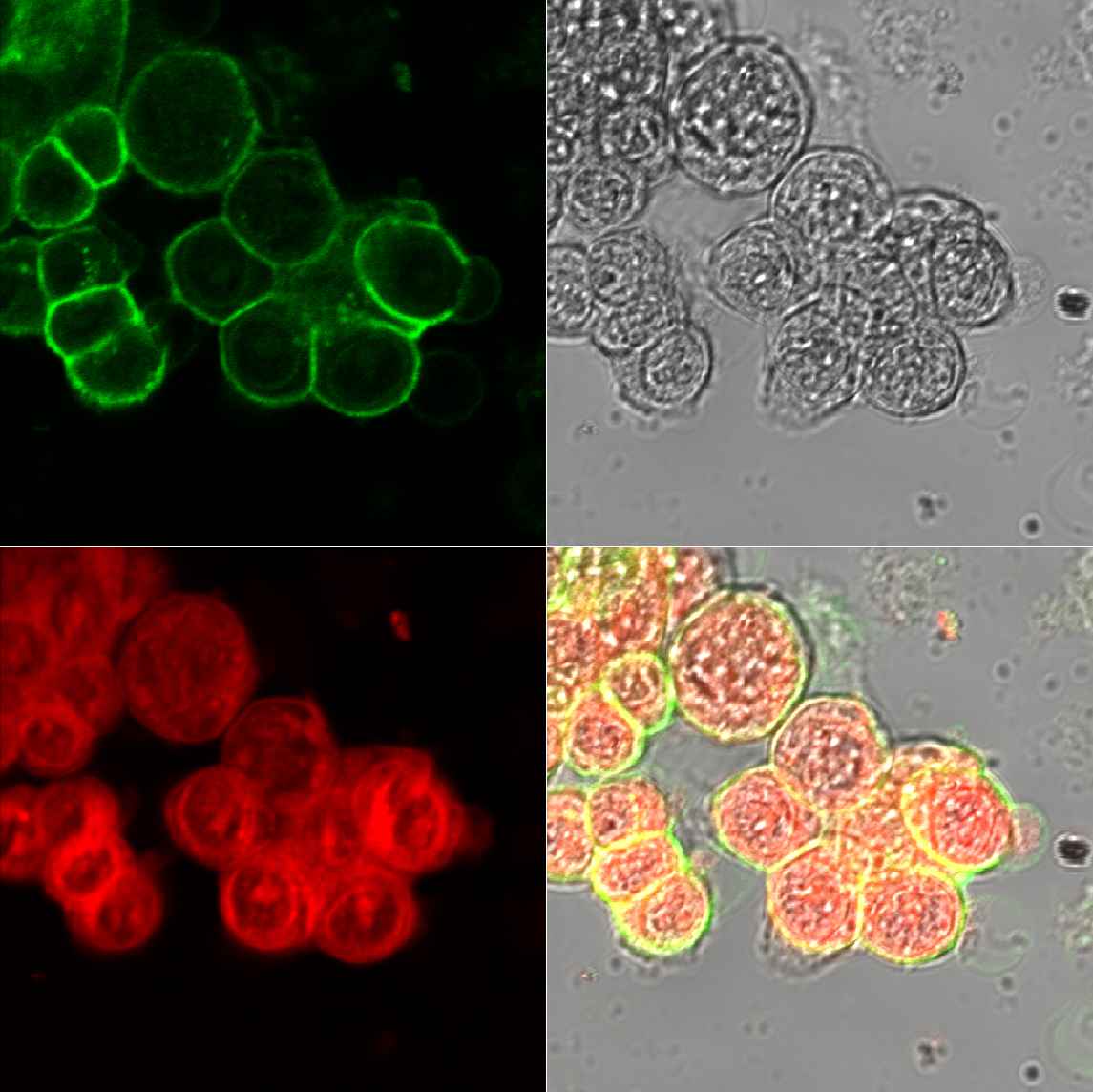


10 um

WGA

Mitotracker Red

Transmitted Image

Merged

**Figure S8**. The HCT116 cells in well 5 viewed under the laser confocal microscope at 100x magnification stained with WGA (green) and mitotracker (red). All of the HCT116 cells imaged here have visibly changed shape and inner configuration during apoptosis when compared to viable HCT116 cells. There is no longer a non-staining area where the nucleus is, however a faint WGA stain suggests existence of permeable nuclear membrane. The membrane that encloses apoptotic body (indicated by arrow is also visible.


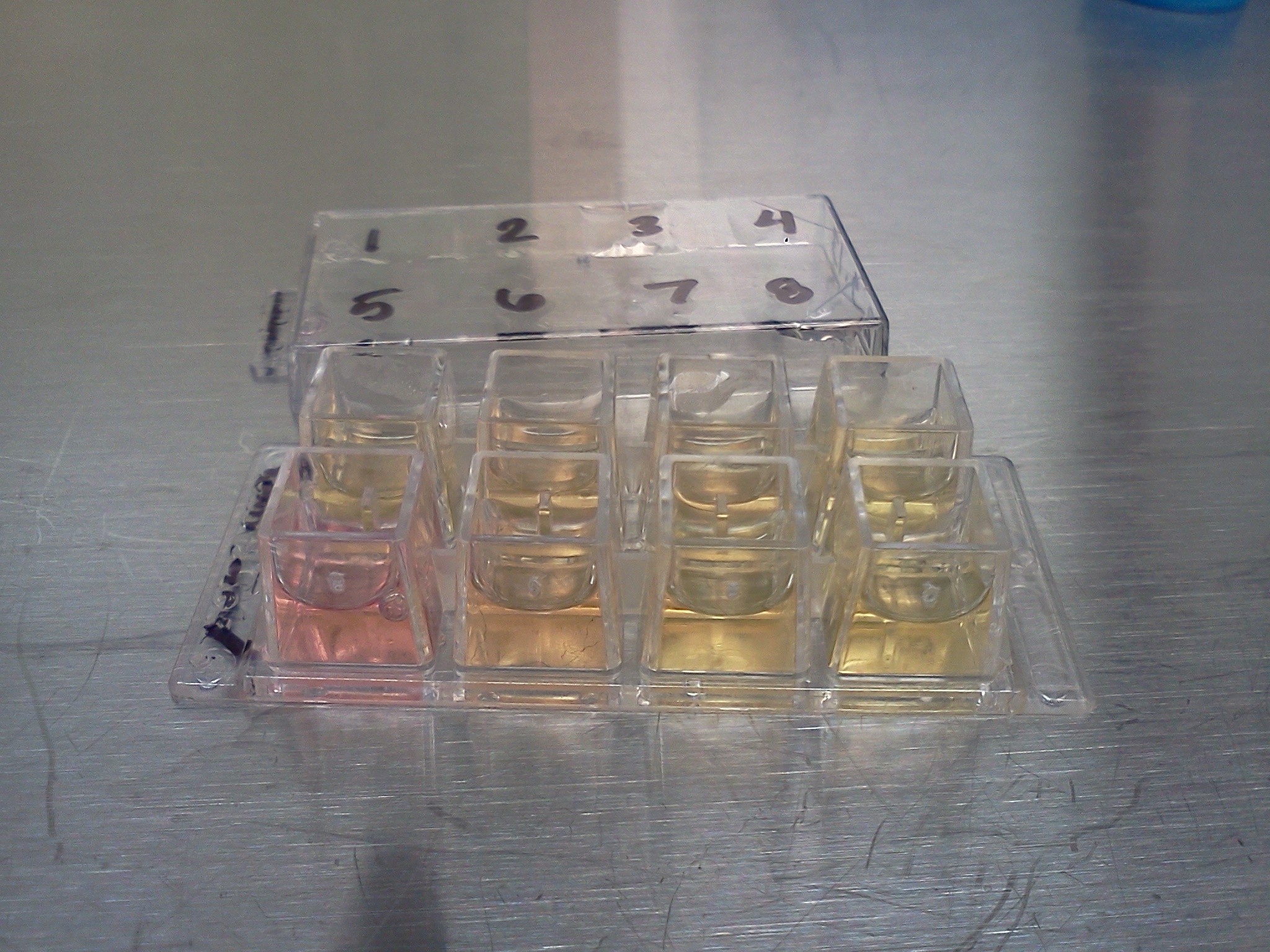


**Figure S9.** A photograph shows macroscopic changes of cellular growth medium color change in the experimental wells of the plate shown in Figure S6. The color of unused growth medium is a pink due to the presence of phenolphthalein red at pH 7, however due to acidification after cells have harvested the nutrients and released the waste products the color changes to a yellow. In the well 5 the growth medium remains pink because the cell cultures are dead and no longer using nutrients or producing waste products. In the well 6 (partially conjugated TiO_2_ and luciferin) there is a slight pink color as some cells have died, but a small concentration are living and using nutrients. In the remaining wells the growth medium has had its nutrients used up by the living cells and is therefore visibly yellow in color.


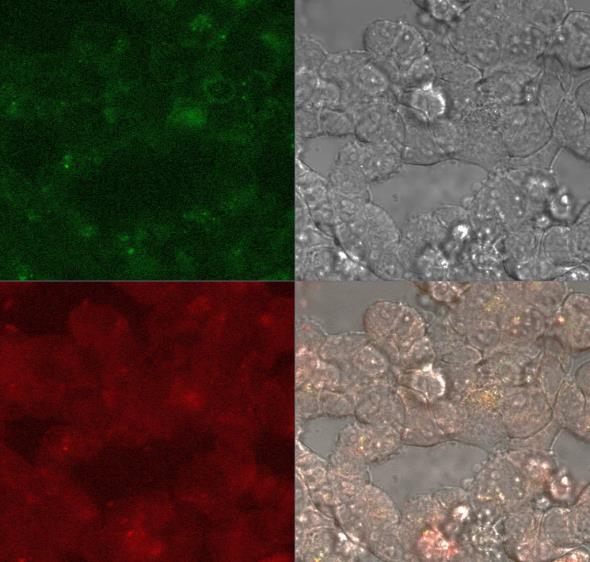

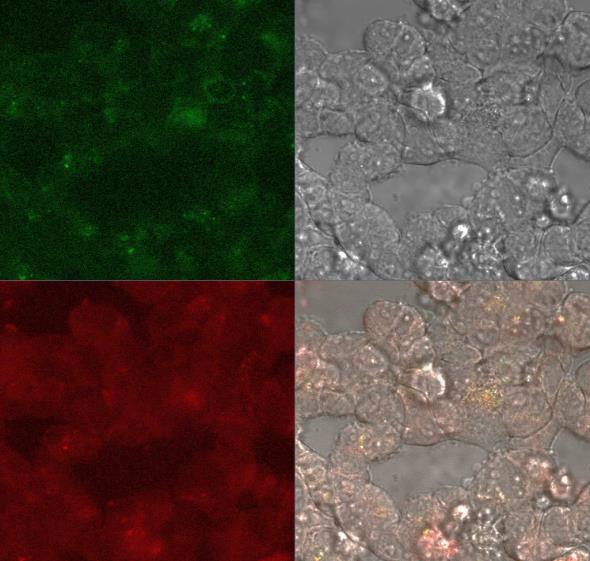


*HCT116*

*TiDoL-Alizarine*

**Figure S10.** Adsorption of TiDoL nanoconjugates in the absence of C225 antibody on EGFR+ HCT116 cells. Nanocomposites are visualized by imaging fluorescence of alizarin bound to TiO2 nanoparticles using laser excitation 560 nm are shown in red, while those imaged in transmission mode are shown in gray. Magnification 40x.

***CT26***


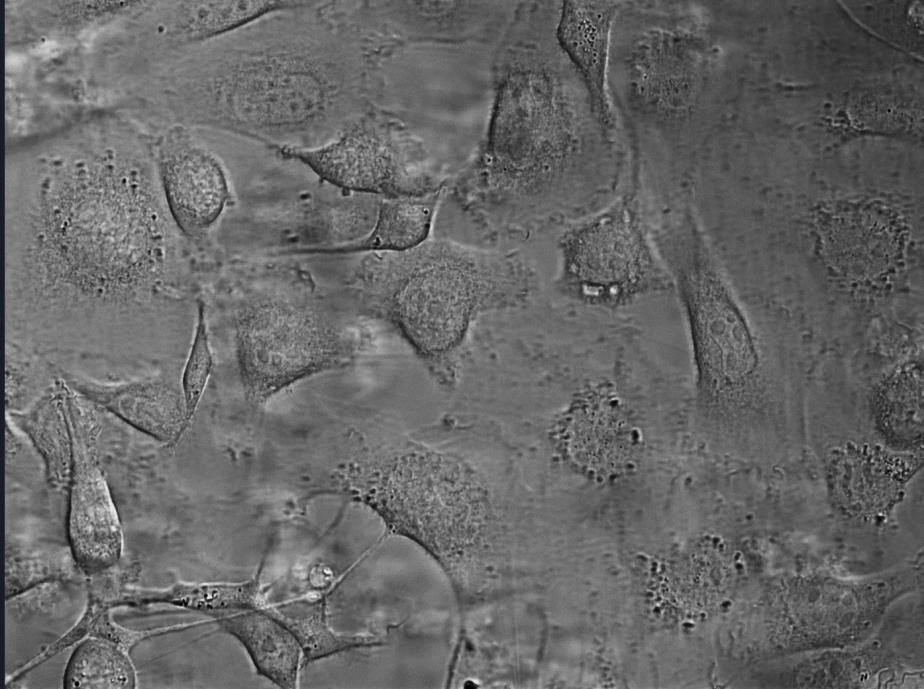

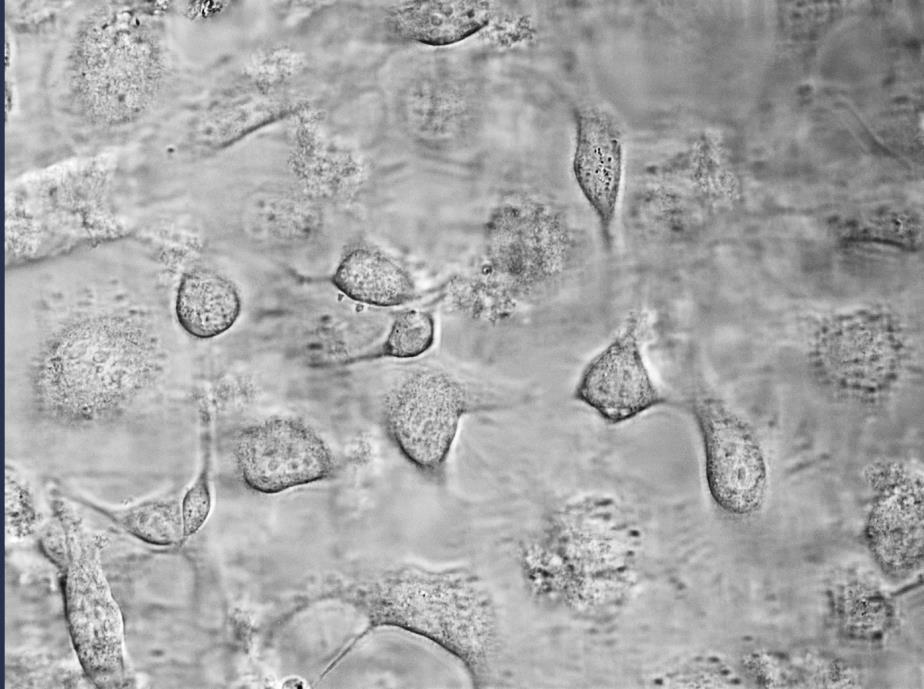

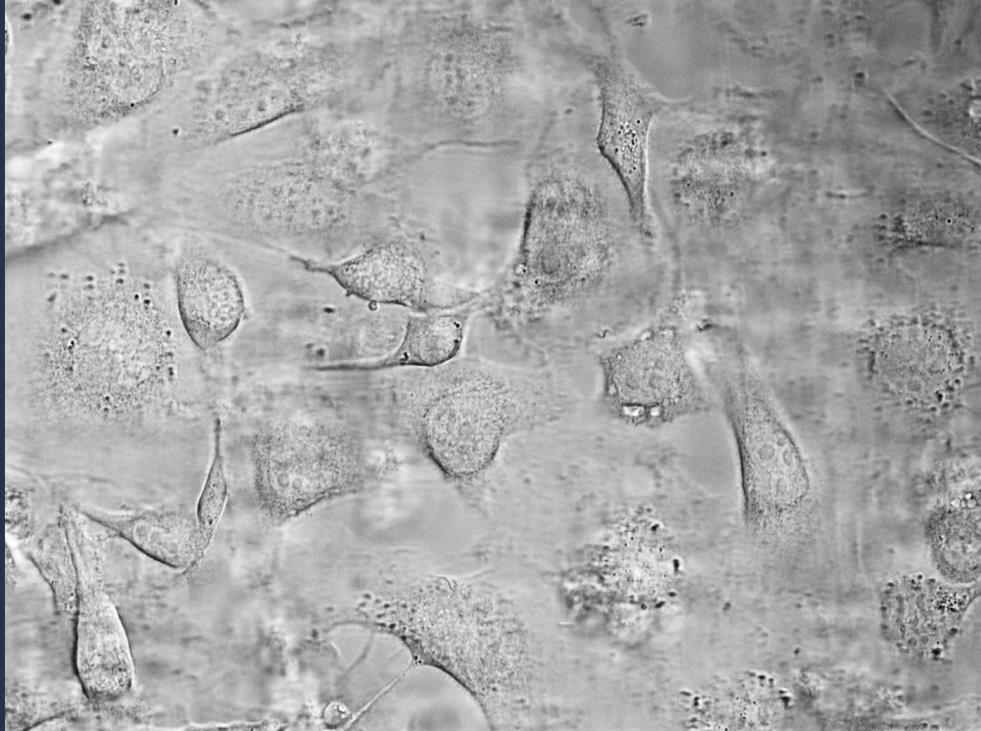


1^st^

0 min

10 min

60 min

***HCT116***

***HCT116***

***CT26***


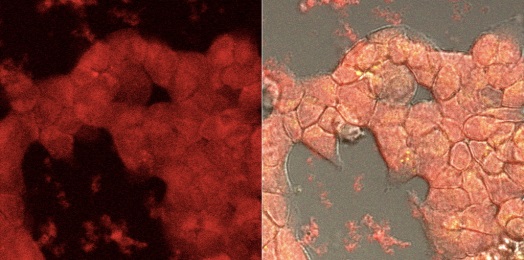

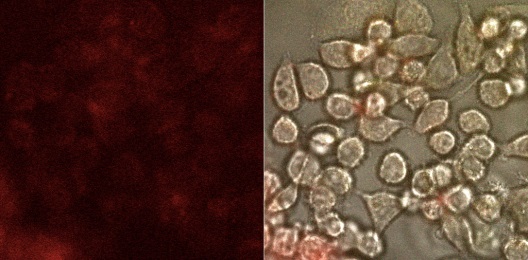

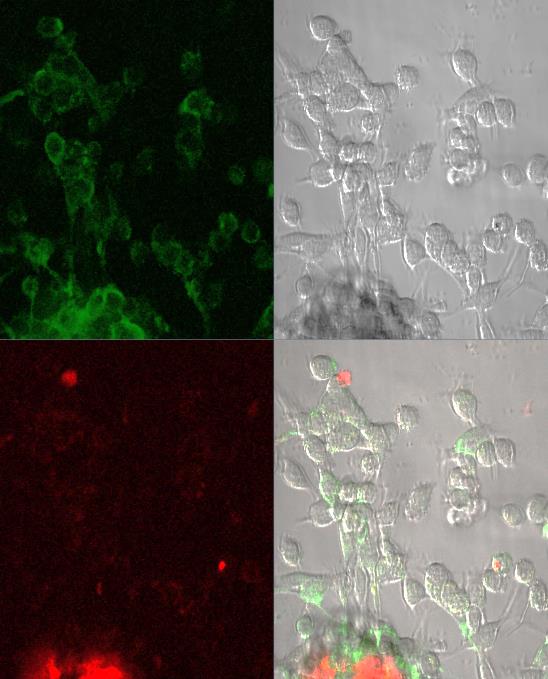


20 μm

*TiDoL-Alizarine-C225*

*TiDoL-Alizarine-C225*

*TiDoL-Alizarine*

Merged

**CT26**

**HCT116**

**HCT116**

TiDoL-C225-alizarin

Merged

**Figure S11. (A)** Time course of the morphological changes of CT26 fibroblast cell line treated with 150 nM TiDoL-C225. Magnification 126x. **(B)** Adsorption of TiDoLC225 nanoconjugates on EGFR+ HCT116 and EGFR- CT26 fibroblast cells. Nanocomposites are visualized by imaging fluorescence of alizarin bound to TiO2 nanoparticles (red) using laser excitation 560 nm while cells are imaged in transmission mode are shown in gray. Magnification 40x.

**Figure S12**. Bar chart of time of budding of HCT116 cells treated by different nanoparticles and activated by luciferin. Time of budding is taken as an indicator of cell death.

TiDoL-C225-alizarin

Merged

Figure S13. (A-D) Laser-scanning confocal fluorescence microscope images of A172 glioblastoma cells before and after treatment with 1.7 µM TiDoL nanoparticles with and without luciferin for 24 hours at 60x magnification. Green color corresponds to cell membrane stained by wheat germ agglutinin and red color corresponds to superoxide radical stained by Mitosox dye. (E) Flow cytometry analysis of A172 cells treated with 1.7 µM TiDoL nanoparticles for 24 hours. As a control the TiDoL nanoparticles were tested both with and without the presence of luciferin. Free luciferase and luciferin without TiO2 nanoparticles, luciferin alone, and PBS treated cells were also used as negative control treatment conditions.
